# Revealing catalyst restructuring and composition during nitrate electroreduction through correlated operando microscopy and spectroscopy

**DOI:** 10.1038/s41563-024-02084-8

**Published:** 2025-01-24

**Authors:** Aram Yoon, Lichen Bai, Fengli Yang, Federico Franco, Chao Zhan, Martina Rüscher, Janis Timoshenko, Christoph Pratsch, Stephan Werner, Hyo Sang Jeon, Mariana Cecilio de Oliveira Monteiro, See Wee Chee, Beatriz Roldan Cuenya

**Affiliations:** 1 Department of Interface Science, Fritz-Haber Institute of the Max-Planck Society, Berlin, Germany; 2 Department of X-ray Microscopy, Helmholtz-Zentrum Berlin, Berlin, Germany; 3 Present Address: Shell Global Energy Solution International BV, Amsterdam, Netherlands; 4 Present Address: Department of Chemical and Pharmaceutical Sciences, University of Trieste, Trieste, Italy; 5 Present Address: College of Chemistry and Chemical Engineering, Xiamen University, Xiamen, China; 6 Present Address: Technological Convergence Center, Korea Institute of Science and Technology (KIST), Seoul, Republic of Korea

**Keywords:** Electrocatalysis, Electrocatalysis, Imaging techniques, Transmission electron microscopy

## Abstract

Electrocatalysts alter their structure and composition during reaction, which can in turn create new active/selective phases. Identifying these changes is crucial for determining how morphology controls catalytic properties but the mechanisms by which operating conditions shape the catalyst’s working state are not yet fully understood. In this study, we show using correlated operando microscopy and spectroscopy that as well-defined Cu_2_O cubes evolve under electrochemical nitrate reduction reaction conditions, distinct catalyst motifs are formed depending on the applied potential and the chemical environment. By further matching the timescales of morphological changes observed via electrochemical liquid cell transmission electron microscopy with time-resolved chemical state information obtained from operando transmission soft X-ray microscopy, hard X-ray absorption spectroscopy and Raman spectroscopy, we reveal that Cu_2_O can be kinetically stabilized alongside metallic copper for extended durations under moderately reductive conditions due to surface hydroxide formation. Finally, we rationalize how the interaction between the electrolyte and the catalyst influences the ammonia selectivity.

## Abstract (web summary)

Studies based on correlated operando characterization techniques reveal the coexistence of copper metal, oxide and hydroxide phases during the electrochemical reduction of nitrates to ammonia, providing insights into electrocatalyst evolution during reaction and related catalytic performance.

## Main

Electrocatalytic chemical conversion reactions such as the carbon dioxide reduction reaction (CO_2_RR)^1,2^ and the nitrate reduction reaction (NO_3_RR)^3,4^ are key to the advancement of various green energy solutions. However, it can be difficult to identify the active catalyst species in these reactions, even when the metallic state is supposed to be the stable phase, because the catalyst can change its oxidation state during reaction according to external stimuli. Although Pourbaix diagrams^5^ can be used to rationalize the stable oxidation state/phase at different applied potentials and pHs, they are equilibrium diagrams which do not consider the kinetics of redox transitions and their effect on catalyst morphology. For example, they do not include information about how oxide-to-metal transformations occur, how different facets can reconstruct differently under the same reaction conditions, how interactions between the catalysts and the electrolyte can alter catalyst surface, or how reaction intermediates and products may lead to further changes.

The challenge here is twofold. First, one must elucidate the working morphology of the electrocatalyst. Second, one needs to disentangle the impact the observed morphological changes have on catalytic performance. There are only a few methods^6,7^ that can visualize the nanoscale restructuring dynamics of a catalyst and follow the catalyst as a function of the applied potential and electrolyte conditions. It is even more challenging to resolve the local chemical state of these features because most operando techniques for extracting chemical information, such as Raman spectroscopy and X-ray absorption spectroscopy (XAS), are ‘broad beam’ methods, where the data are an ensemble signal derived from a large probed region. This gap between nanoscale imaging and ensemble-averaging spectroscopy limits our ability to rationalize how catalyst morphology impacts the overall performance of these complex but important reactions.

The NO_3_RR is noteworthy among the various electrochemical conversion reactions in terms of the questions regarding its working electrocatalyst phase during the reaction. Foremost, this reaction offers a promising strategy for mitigating freshwater pollution from agricultural fertilizer run-off and industrial waste^8^, and has also been studied for its potential to produce NH_3_ (refs. ^9–11^), an important industrial chemical and a candidate carrier for green hydrogen^12,13^. Although copper is one of the most-studied electrocatalyst materials for the NO_3_RR due to its optimal nitrate adsorption energy^11,14^, whether metallic copper^15–18^, copper oxides or a Cu–Cu oxide interface^19^ are the key species for selective NH_3_ formation has remained largely unresolved. According to the Pourbaix diagram^20,21^, the metallic phase of copper should be the stable phase under typical NO_3_RR conditions, but studies using in situ Raman spectroscopy suggested that an oxide phase might exist during the reaction^14,19^. Copper and its oxides are also known to be susceptible to etching^22^ and facet modification^23,24^ by NH_3_. Furthermore, it has been reported that the NO_3_RR can drive the dissolution and regrowth of single-atom copper catalysts^3^, and the clustering of small aggregates into larger nanoparticles (NPs)^25^.

In this study, we use electrochemical liquid cell transmission electron microscopy (EC-TEM) accompanied by correlated multimodal operando investigations that include electrochemical liquid cell transmission X-ray microscopy (EC-TXM), operando XAS and operando Raman spectroscopy of the same precatalysts to visualize in real time how the structure and composition of Cu_2_O cubes evolve as a function of the applied potential during the NO_3_RR. We found that the working electrocatalyst morphology was determined by three processes: (1) the dissolution of Cu_2_O, (2) the redeposition of copper from soluble copper complexes^26,27^ and (3) the reduction of Cu_2_O to metallic copper. We also discovered a coexistence of Cu_2_O with metallic copper for extended reaction durations, thereby providing insight into the copper species active during the NO_3_RR.

**Fig. 1:**
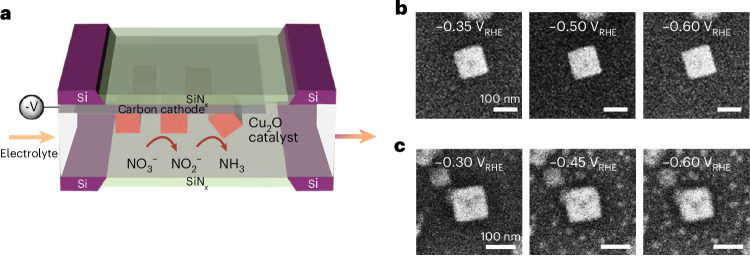
Differences in the restructuring of Cu_2_O catalysts between the NO_3_RR and the CO_2_RR during initial linear sweep voltammetry scan. **a**, Schematic of the EC-TEM experimental configuration in which the Cu_2_O precatalyst was electrodeposited on the working electrode of an EC-TEM chip prior to the experiment. **b**,**c**, Snapshots showing the restructuring of Cu_2_O cubes as observed by operando EC-TEM during linear sweep voltammetry under NO_3_RR conditions in 0.1 M Na_2_SO_4_ + 8 mM NaNO_3_, pH 7 (Supplementary Video 1) (**b**) and an image sequence describing the behaviour of identically synthesized cubes under CO_2_RR conditions in CO_2_-saturated 0.1 M KHCO_3_, pH 6.8 at a similar applied potential range (Supplementary Video 2) (**c**). The electron flux used in these experiments is 1.75 *e*^−^ A^−2^ s^−1^.

For the operando microscopy experiments, we prepared well-defined Cu_2_O cubes on the carbon working electrode of the EC-TEM chips via electrodeposition^28,29^ as shown schematically in Fig. 1a. The as-prepared Cu_2_O cubes have an average size of 250 nm and consist of six {100} facets without the exposure of other minor facets such as {110} or {111} (Supplementary Fig. 1). All voltages indicated in this paper are referenced against a Ag/AgCl electrode and then converted to the reversible hydrogen electrode scale (RHE) using the Nernst equation and the bulk pH of the electrolyte. Intriguingly, the image sequences show that the cubes do not undergo obvious change in a typical 0.1 M Na_2_SO_4_ + 8 mM NaNO_3_ electrolyte for the NO_3_RR (Fig. 1b) during the initial potential sweep towards cathodic potentials. According to the Pourbaix diagram, the redox potential for the transformation of Cu_2_O to metallic copper is 0 V_RHE_ in a solution with pH 7 (refs. ^20,21^) and metallic copper is the stable phase below −0.2 V_RHE_ onwards, and therefore Cu_2_O should reduce directly to metallic copper at the higher overpotentials of the sweep. The stability of the Cu_2_O cubes is remarkable because these redox transformations usually lead to morphological changes. For comparison, Fig. 1c depicts a Cu_2_O cube under CO_2_RR conditions in CO_2_-saturated 0.1 M KHCO_3_ at a potential similar to that applied in the NO_3_RR experiment. As we reported previously^29^, the latter cubes undergo fragmentation together with the redeposition of small particles, a behaviour that differs from the morphologically much more stable NO_3_RR samples at the same applied potentials. The linear sweep voltammograms acquired during these two experiments are shown in Supplementary Fig. 1.

**Fig. 2:**
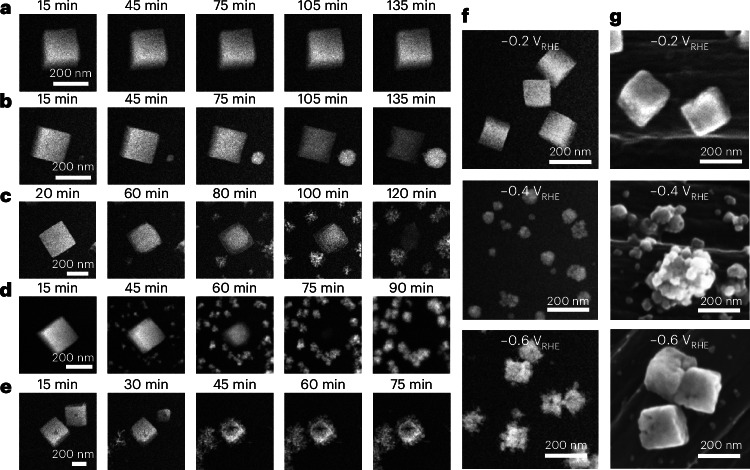
Potential- and time-dependent restructuring of Cu_2_O cubes observed with EC-TEM. **a**–**e**, Operando EC-TEM time series of Cu_2_O cube restructuring acquired with intermittent imaging at −0.2 V_RHE_ (**a**), −0.3 V_RHE_ (**b**), −0.4 V_RHE_ (**c**), −0.5 V_RHE_ (**d**) and −0.6 V_RHE_ (**e**) during the reaction at the indicated times in 0.1 M Na_2_SO_4_ + 8 mM NaNO_3_. A new sample was used at each applied potential. The electron flux used in these experiments is 1.75 *e*^−^ A^−2^ s^−1^. **f**,**g**, Comparison of postreaction images of in situ and ex situ experiments: EC-TEM images after 2 h at each designated potential (**f**), and SEM images of Cu_2_O cubes electrodeposited on carbon paper and reacted on the benchtop for 2 h at the same applied potentials (**g**).

Next, we studied these Cu_2_O cubes systematically at different sustained potentials from −0.2 V_RHE_ to −0.6 V_RHE_ (Fig. 2a–e) to probe further their morphological stability during the NO_3_RR. For these extended experiments, we adopted an intermittent imaging protocol (in which images were captured at 15 min intervals with the electron beam blanked the rest of the time) to minimize beam-induced dissolution of the Cu_2_O cubes (see discussion in Supplementary Note 1) and ensure that the catalyst restructuring kinetics we extract from the collected data are as accurate as possible. The electrochemical current profiles over time at each potential measured in these EC-TEM experiments are shown in Supplementary Fig. 3. At −0.2 V_RHE_ (Fig. 2a), the cubes were stable during our entire observation, with no notable restructuring observed. From −0.2 to −0.5 V_RHE_, dissolution/redeposition is the main restructuring pathway. At −0.3 V_RHE_, the cubic form persisted for almost 135 min (Fig. 2b), while the cube completely dissolved after 140 min at −0.4 V_RHE_ (Fig. 2c) and after 90 min at −0.5 V_RHE_ (Fig. 2d). The lighter contrast of the cube exterior in Fig. 2c compared with the middle of the cube at 60 and 80 min is explained by the cube corners and edges being etched first. The weaker contrast of the dissolving Cu_2_O cubes compared with that of the growing Cu NPs also suggests that the dissolving cubes were still in oxide form.

Two Cu_2_O cubes were captured in the images acquired at −0.6 V_RHE_ (Fig. 2e). One cube shrank and restructured into a smaller cube with a void in the centre and then became rougher due to small NPs attaching to its surface, while another completely dissolved within the same time frame. We also highlight that the intensity of the cubes in the TEM images obtained from −0.3 to −0.5 V_RHE_ gradually decrease, whereas the intensity of the cube at −0.6 V_RHE_ is brighter, implying that the cube-like frame at −0.6 V_RHE_ is metallic. Moreover, the interplay of dissolution/redeposition and direct reduction at the more cathodic potentials means that the terminal catalyst morphologies of oxide precatalysts vary depending on the applied potential.

Next, we repeated the NO_3_RR experiments in an H-type cell with Cu_2_O cubes electrodeposited on carbon paper to compare the consistency of the EC-TEM results versus standard reaction geometries. Figure 2f,g shows lower-magnification images of samples from the EC-TEM experiments with scanning electron microscopy (SEM) images of samples extracted from H-type cell experiments after 2 h of reaction at three different applied potentials, −0.2, −0.4 and −0.6 V_RHE_. Electron diffraction patterns taken from samples extracted after the reaction show that the cubes did not undergo extensive restructuring at −0.2 V_RHE_ and remain Cu_2_O, whereas samples reacted at −0.6 V_RHE_ were largely metallic (Supplementary Fig. 4). Conversely, samples reacted at −0.4 V_RHE_ show a mixture of residual Cu_2_O and metallic copper structures (Supplementary Figs. 4–6). The morphological differences between the sample after reaction in the H-type cell at −0.4 and −0.6 V_RHE_ further support that the catalysts indeed restructure through different pathways as described by our EC-TEM experiments. Inductively coupled plasma mass spectrometry measurements of the electrode and the electrolyte in the H-type cell after reaction also show that copper dissolution occurs at all the applied potentials (Supplementary Fig. 7). Therefore, these experiments indicate that the Cu_2_O cubes undergo a gradual dissolution under NO_3_RR conditions, which in turn leads to the redeposition of metallic particles elsewhere on the working electrode with shapes and sizes that are modulated by the applied potential.

**Fig. 3:**
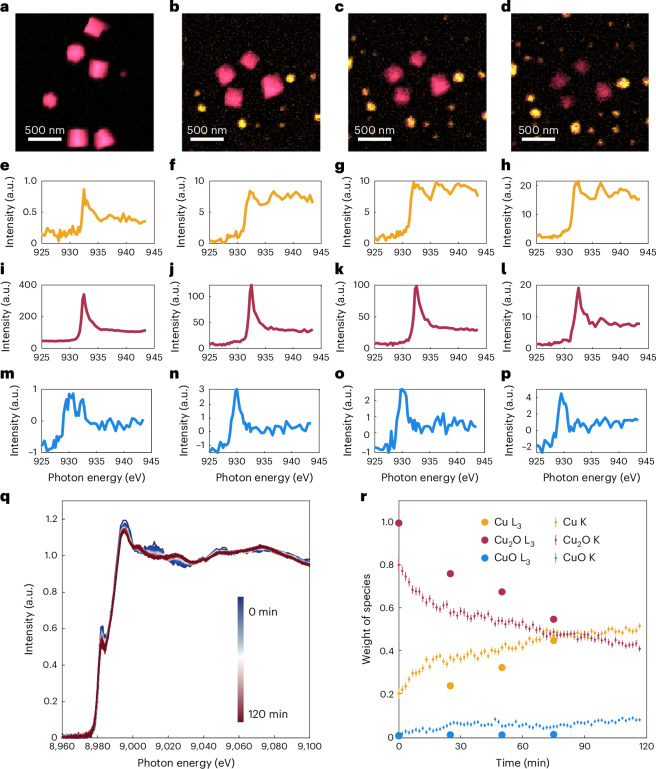
Correlating the morphological evolution with changes in the chemical state of the Cu_2_O precatalysts during the NO_3_RR with operando TXM and XANES measurements. **a**–**d**, Spatially and temporally evolving copper catalysts and their valence states as observed with TXM. Coloured map of Cu_2_O cubes and redeposited copper catalysts before reaction in their original dry state (**a**), and after 25 min (**b**), 50 min (**c**) and 75 min (**d**) of TXM acquisition at −0.4 V_RHE_ in 0.1 M Na_2_SO_4_ + 8 mM NaNO_3_. **e**–**p**, Copper L_3_-edge XAS intensity extracted from the TXM image stacks in **a**–**d**, integrated over from individual pixels in the coloured areas of the images and then decomposed into three components where the coloured spectra correspond to copper (yellow) (**e**–**h**), Cu_2_O (red) (**i**–**l**) and CuO (blue) (**m**–**p**); original state (**e**,**i**,**m**), and after 25 min (**f**,**j**,**n**), 50 min (**g**,**k**,**o**) and 75 min (**h**,**l**,**p**). **q**, Copper K-edge XANES measured at −0.4 V_RHE_ in 0.1 M Na_2_SO_4_ + 8 mM NaNO_3_. The colour bar denotes the acquisition time of the respective XAS spectra from the start of the measurements. **r**, Temporal evolution in the weights of copper, Cu_2_O and CuO species obtained by fitting the copper L_3_ and K edges obtained from operando TXM and operando XANES, respectively. The error bars in the XANES dataset refer to the standard errors of the fitting procedure.

To obtain unambiguously the oxidation state of the catalyst species present during reaction and rule out the possibility that the ex situ identified Cu_2_O phase is the result of reoxidation during the return to open circuit potential^30,31^ (OCP), we performed operando EC-TXM measurements on the Cu_2_O cubes by transferring our EC-TEM holder into a TXM at the BESSY II synchrotron facility as illustrated in Supplementary Fig. 8. This unique arrangement maintains the same reaction environment between the two experiments, while enabling time-resolved operando measurements of copper absorption edges under applied potential without compromising the sustained electrolyte flow because X-rays are attenuated less by the electrolyte and enclosing membranes than electrons. Thus, the evolution of the electrocatalysts’ composition can be tracked during the NO_3_RR. Figure 3a–d shows the time-resolved evolution of the Cu_2_O catalysts during the NO_3_RR at −0.4 V_RHE_ as observed by EC-TXM in the form of the coloured maps that were reconstructed from a XAS image stack using linear combination fitting (LCF)^32^. Cu_2_O is depicted in red and metallic copper in yellow. The maps show that Cu_2_O and copper are the dominant phases present for the duration of the NO_3_RR and that the oxide and metallic phases coexist under specific reaction conditions, but are spatially separated. CuO is also detected (blue) but is present only in small quantities and is not clearly visible from the maps. The corresponding decomposed spectra are shown in Fig. 3e–p, where the yellow, red and blue lines represent the respective copper species. The total contribution of the individual spectra in Fig. 3e–p represents the amount (thickness) of each species, which indicates that the content of metallic copper species increases (Fig. 3e–h) during the reaction, whereas Cu_2_O decreases (Fig. 3i–p). Details of the data acquisition and processing are discussed in Supplementary Note 3. Most importantly, these results confirm the sluggish reduction kinetics of the large Cu_2_O cubes in the Na_2_SO_4_ + NaNO_3_ electrolyte, and that the metallic phase forms when the dissolved copper species redeposit on the working electrode due to the reductive potential employed.

We further verified that the slow reduction of the copper oxide cubes extends to larger reaction volumes with operando hard XAS measurements of samples electrodeposited on carbon paper in our home-built electrochemical XAS cell^33^. In Fig. 3q, we plot the changes in the copper K-edge valence states (from 8,950 to 9,105 eV) that were obtained from operando XAS. The weight of the copper valence state is extracted by LCF of the X-ray absorption near-edge structure (XANES) of the oxide-derived copper catalyst collected at a constant potential of −0.4 V_RHE_ in 0.1 M Na_2_SO_4_ + 8 mM NaNO_3_ electrolyte. As seen in Fig. 3r, the fraction of Cu_2_O decreased, but did not completely disappear, after more than 2 h of electrolysis, while the fraction of metallic copper increased correspondingly, eventually to almost a 1:1 ratio of Cu_2_O:Cu. The XANES results agree with the persistence of Cu_2_O and the continual evolution of the copper species seen in the EC-TEM (Fig. 2c) and EC-TXM (Cu L_3_ edges in Fig. 3r) results at −0.4 V_RHE_. Minute amounts of the CuO species were also detected during the experiment. The changes in the weights of the three species over time exhibit similar trends in both TXM and XAS, confirming that the results we obtain in the EC-TEM cells indeed extrapolate to a larger ensemble of catalyst particles.

**Fig. 4:**
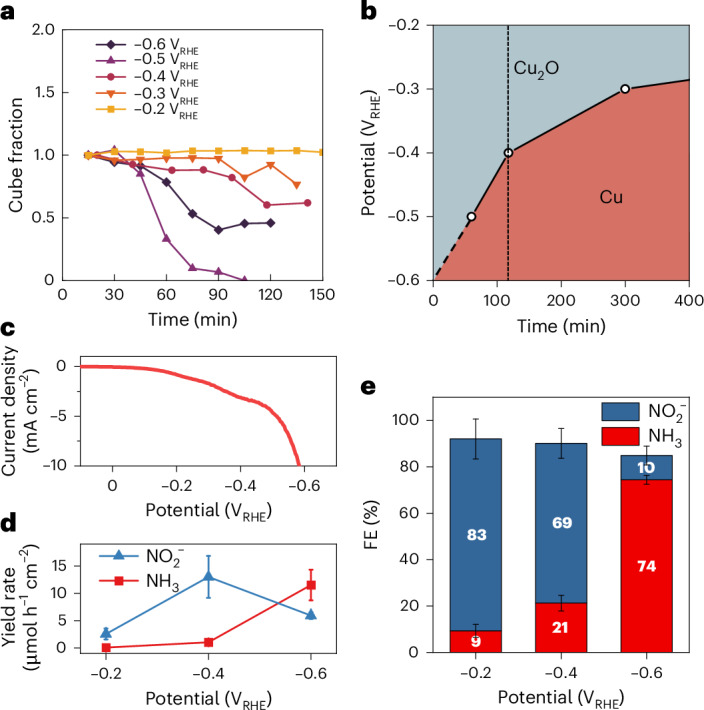
Temporal evolution of oxidic and metallic copper phases and its impact on NH_3_ selectivity. **a**, The fractions of the cubes within the EC-TEM images are calculated by dividing the area of the reacted cubes at time *t* by the initial projected area of Cu_2_O precatalysts at time 0. **b**, Plot depicting the majority copper phase at different applied potentials and reaction times. The solid line indicates the estimated time to reduce the size of a Cu_2_O cube by 50%. The vertical dashed line denotes 2 h of the NO_3_RR. The diagonal dash line extrapolates from 60 min at –0.5 V_RHE_ to 0 min at –0.6 V_RHE_ as the first image acquired at 15 min in the –0.6 V_RHE_ experiment already indicates cube reduction. **c**, Linear sweep voltammetry of electrodeposited Cu_2_O cubes on carbon paper measured from 0.1 to −0.6 V_RHE_. **d**,**e**, The yield rate of NO_2_^−^ and NH_3_ (**d**) and the FE of NO_3_RR products (**e**) at −0.2, −0.4 and −0.6 V_RHE_. The error bars in **d**,**e** indicate the s.d. of three independent measurements.

This overall agreement between different methods and experimental geometries means that we can use the in situ TEM image sequences to quantify the potential-dependent dissolution and redeposition rates. Our method for fraction extraction from the EC-TEM images and additional analysis of the redeposited particles is described in Supplementary Note 4. As shown in Fig. 4a, the cube fraction decreases over time at an increasing rate as the potential decreases from −0.2 to −0.5 V_RHE_. The sample at −0.6 V_RHE_ deviates from this trend (dark purple line) because of the direct reduction of Cu_2_O to metallic copper. In Fig. 4b, we use the cube dissolution rate to estimate the Cu_2_O:Cu ratio at a certain potential and use it to visualize the majority phase (>50%) at different times. We further compare the Cu_2_O:Cu ratio with NH_3_ conversion activity (current density) and selectivity (Faradaic efficiency (FE)) obtained from our benchtop electrochemistry measurements. In Fig. 4c, we plot the linear sweep voltammogram of the Cu_2_O cubes prepared on carbon paper, and in Fig. 4d,e, we show their product distribution as a function of the applied potentials. The measured yield rate and the FE towards NH_3_ were much higher at −0.6 V_RHE_ than at −0.2 and −0.4 V_RHE_, implying that the change in catalytic selectivity is related to the faster rate of oxide to metal conversion at −0.6 V_RHE_ (Fig. 4b).

Next, we performed EC-TEM studies in various electrolyte compositions to elucidate the mechanism behind Cu_2_O stabilization. Supplementary Fig. 9a,b describes experiments using pure 0.1 M Na_2_SO_4_ and 0.1 M Na_2_SO_4_ + 8 mM NaNO_2_ (that is, nitrite reduction), respectively. In both cases, the cubes behaved similarly to their behaviour in the NO_3_RR, during which they gradually reduced in size until they fragment/reduce at longer reaction durations, which means the Cu_2_O stability is related to the Na_2_SO_4_ supporting electrolyte and not the reactant. Redeposition was, however, much less in Na_2_SO_4_ than in its NaNO_3_/NaNO_2_-containing counterparts. We attribute this difference to how the local pH during electroreduction differs in the presence and absence of NO_*x*_ species. Under applied cathodic potentials, the pH at the electrocatalyst surface increases as hydroxyl ions form^34,35^ due to the reduction of, for example, H_2_O, O_2_, NO_2_^−^ and NO_3_^−^. In particular, the NO_*x*_RR results in higher currents and consequently a steeper rise in the local pH compared with when only hydrogen reduction takes place. This process can bring the pH of a neutral electrolyte to above 12 (ref. ^34^), triggering the formation of soluble copper hydroxides. To investigate the effect of electrolyte pH, we further performed experiments in 0.1 M Na_2_SO_4_ where the pH was increased to 10 by adding NaOH. As shown in Supplementary Fig. 9c, this altered the amount of redeposition observed. Finally, to probe the influence of ammonium ions, we deliberately added NH_4_OH to the 0.1 M Na_2_SO_4_ carrier electrolyte, which led to rapid restructuring of the cubes as shown in Supplementary Fig. 9d.

To explain these results, we consider the phase stability of copper as a function of pH and in the presence of NH_3_. A complex series of reactions encompassing different acid–base chemistries, Cu(OH)_2_ precipitation and complex ion formation are known for the Cu–NH_3_ system^36,37^ (Supplementary Note 5). Specifically, the equilibrium between solid Cu(OH)_2_ and the Cu(NH_3_)_4_^2+^ complex depends on the NH_3_ concentration, with Cu(OH)_2_ precipitation being favoured at low concentrations due to the poor solubility of Cu(OH)_2_ (ref. ^36^). We hypothesize that the sluggish reduction observed may be the result of transient surface Cu(OH)_2_ formation induced by the interfacial pH rise in the course of the NO_3_RR. Cu(OH)_2_ formation may also be more favourable in Na_2_SO_4_, due to the electrolyte’s inability to buffer the increase in local pH from electroreduction^34^, as compared to the KHCO_3_ electrolyte used in CO_2_RR, thereby leading to the differences in restructuring behaviours. To validate this hypothesis, we studied the chemical changes taking place on the surface of the Cu_2_O cubes with operando Raman spectroscopy measurements.

**Fig. 5:**
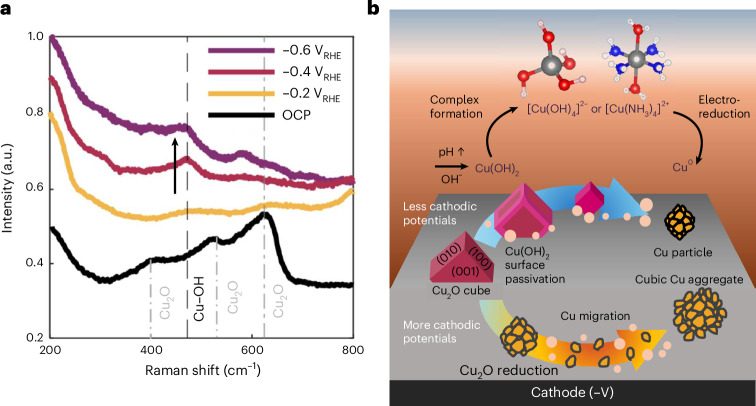
Probing surface chemistry changes in Cu_2_O cubes using operando Raman spectroscopy and schematic detailing catalyst restructuring mediated by oxide/hydroxide formation. **a**, Operando Raman spectra of Cu_2_O cubes electrodeposited on glassy carbon measured during the NO_3_RR in 0.1 M Na_2_SO_4_ + 8 mM NaNO_3_ at OCP, −0.2 V_RHE_, −0.4 V_RHE_ and −0.6 V_RHE_. The average of the 12 measurements is plotted. The arrow highlights the peak at ~475 cm^-1^. The measurements were conducted with a fresh specimen for each potential. **b**, Schematic describing the possible restructuring mechanisms depending on the applied cathodic potential and how the Cu(OH)_2_ ⇌ Cu(OH_4_)^2−^/Cu(NH_3_)_4_^2+^ equilibrium may be controlling the dissolution/redeposition process.

Figure 5a shows the results of experiments at constant applied potentials performed with cubes electrodeposited on glassy carbon plates. At OCP, the Raman spectrum shows three features of bands centred at 415, 520 and 630 cm^−1^, respectively, which are in good agreement with the reported values of Cu_2_O (refs. ^38,39^). When −0.2 V_RHE_ was applied, the band intensity at 520 and 630 cm^-1^ decreased over time but continued to persist, which is consistent with the gradual dissolution of Cu_2_O. At −0.4 V_RHE_, a new peak at 475 cm^−^^1^ started to emerge, while the peak at 630 cm^−^^1^ flattened, indicating oxide to metal transition. At −0.6 V_RHE_, the characteristic bands of Cu_2_O were less pronounced and additional weak bands emerged at 450, 475 and 590 cm^−^^1^. The band around 475 cm^−^^1^ can be assigned to the Cu–O–H vibration^39–41^ or Cu(OH)_2_ (ref. ^40^), while the peak at 590 cm^−^^1^ is often assigned to CuO (ref. ^38^) or adsorbed oxygen species on copper^41^. These results suggest the formation of a transient intermediate oxide or hydroxide phase during electrolysis, which is also supported by the extended presence of Cu(I) signatures and the weak but persistent Cu(II) signatures in the EC-TXM and operando XAS measurements in Fig. 3. It was, however, difficult to obtain the Raman signatures of absorbates with these samples due to the relatively low loading of the cubes that we were able to electrodeposit and the overlap of the D/G bands of the glassy carbon support with surface absorbate bands, which limits the signal-to-noise ratios at those bands. Thus, we repeated the Raman measurements with cubes electrodeposited at higher loading on carbon paper to improve the signal-to-noise ratios to identify surface adsorbed species or intermediate species in the vicinity of electrode. These results are discussed in Supplementary Note 6.

Hence, we arrive at a restructuring mechanism, illustrated in Fig. 5b, according to which surface hydroxides first form on the Cu_2_O cubes due to the pH increase induced by electrolysis, which delays the oxide reduction. The subsequent NH_3_ production and added pH rise from continued NO_3_RR then destabilize this hydroxide layer and form soluble copper complexes, thereby initiating catalyst evolution via redeposition from copper complex reduction or the aggregation of migrating NPs. We also performed more EC-TEM experiments to check that the delayed restructuring kinetics extend to other precatalyst geometries. Supplementary Fig. 10 describes the evolution during the NO_3_RR of Cu_2_O truncated octahedra and metallic frames created by prereducing the Cu_2_O cubes. Both samples are stable during early-stage reaction. With more time, redeposition similar to that seen in the cubes was observed in the Cu_2_O octahedra, whereas little redeposition was noticeable with the metallic frames, probably due to less dissolution occurring when we start from metallic precatalysts.

The correlated microscopy and spectroscopy experiments presented here therefore indicate that the morphology of the copper catalysts during the NO_3_RR at a given pH is governed by a complex, time- and potential-dependent interplay of three processes: (1) dissolution of oxide and hydroxide species, (2) metal redeposition and (3) oxide catalyst reduction. According to the Pourbaix diagram^20,21^, metallic copper is the only stable species under the specific applied potentials and pH of our experiments, but we have shown that oxidic and metallic phases can coexist over extended durations and over a broad range of applied potentials, which has serious implications in terms of determining the active species for producing NH_3_. It has been suggested previously^19^, based on operando spectroscopy measurements of CuO precatalysts, that Cu/Cu_2_O interfaces are responsible for NH_3_ production^19^, but these methods cannot differentiate the distribution of these species on the nanoscale. As we have shown in this work, the presence of both spectroscopic signatures in ensemble-averaging measurements does not necessarily mean that the two phases are spatially connected. Furthermore, we have demonstrated that the decoupled metallic copper and copper-oxide phases can persist for extended durations at mild cathodic potentials (less than −0.5 V_RHE_), and that a high residual abundance of Cu_2_O in the operando XAS measurements corresponded to a low NH_3_ production efficiency in our electrolysis data of equivalent samples. The improvement of NH_3_ selectivity with increasing overall metallic character of the samples therefore suggests that metallic copper, rather than Cu_2_O, is the active phase for producing NH_3_, in agreement with recent work on the topic^15–18^. In this case, the strong stability of the Cu_2_O cubes and their sluggish reduction kinetics in the often-used Na_2_SO_4_ carrier electrolyte are detrimental to NH_3_ production by delaying the onset of selective NH_3_ formation.

By showing the diverse behaviours that can be elicited in different electrolytes and under a range of reaction conditions, our work also illustrates the critical need to pay attention to how the electrolyte can influence the restructuring of catalysts and the stability of oxide, hydroxide and metallic phases before we attempt to generalize results across different studies and reactions. So far, the description of electrolyte effects in electrolysis has been largely confined to cation adsorption effects^42–45^ and restructuring induced by aggressive halide anions^44–46^, whereas studies of pH had focused on its impact on reaction mechanisms and NH_3_ selectivity^8,10^, and not catalyst phase stability. Substantial additional microscopy work, such as the one presented here, will be required to separate the impact of electrolyte-driven morphological transformation from the much better understood associated electronic and chemical changes. Furthermore, current computational models still cannot rationalize the impact of an explicit complex electrolyte on the catalyst restructuring and its associated influence on the creation of active sites. Efforts to improve these models and advance the theory describing electrocatalytic processes will undoubtedly require more accurate representations of dynamic catalyst surfaces. The challenge here is serious because theoretical mechanistic insight must consider two simultaneously occurring dynamic processes, namely one that the catalyst material undergoes and another that the reactants experiences, both of which are coupled and driven by the local chemical potential^47^. Our results revealing phase coexistence also open the possibility that different species may be responsible for activating specific steps of the conversion reaction (for instance, oxide for NO_3_^–^ to NO_2_^–^ and metal for NO_2_^–^ to NH_3_). Hence, we expect operando approaches that incorporate chemically resolved microscopy within multimodal spectroscopic investigations, as demonstrated here, to play a vital role in moving forward the understanding of electrocatalytic processes by providing a path towards mapping such complexity.

In summary, operando EC-TEM and EC-TXM measurements have revealed that the morphologies of Cu_2_O precatalysts during the NO_3_RR and their evolutionary pathways are sensitive to the reaction time, applied potential and the nature of the electrolyte. As expected, the rate of oxide reduction accelerates with increasing negative applied potentials, but spatially separated oxide and metallic phases can coexist over extended reaction times under moderately reductive potentials. More importantly, the kinetics of the different restructuring processes, which have been unveiled here, determine the final morphology of the catalysts. Our results also indicate that the nature of the electrolyte can introduce time-dependent selectivity changes in the early stages of the catalyst restructuring, which will help resolve ongoing controversies regarding the active state of copper for selective NH_3_ production. Finally, this work impacts our understanding of how electrocatalysts evolve under reaction conditions through the discovery of copper oxide and hydroxide stability. In addition, we revealed local structural and chemical heterogeneities that develop under electrochemical working conditions, even on a precatalyst sample initially characterized by a narrow size, shape and compositional distribution. Thus, our findings emphasize the need for operando characterization methods to establish connections between materials’ structural and compositional characteristics under specific reaction environments and external stimuli and their electrocatalytic performance.

## Methods

### Specimen preparation

The Cu_2_O cubes (250 nm) were synthesized on polished glassy carbon (vitreous, SPI) plates, carbon paper and the carbon electrode of Hummingbird Scientific EC-TEM chips using an electrodeposition protocol we previously developed^28,29^. The deposition solution consists of a mixture of 5 mM copper sulfate pentahydrate (CuSO_4_·5H_2_O, Sigma-Aldrich) and 12.5 mM of potassium chloride (KCl, Sigma-Aldrich). After the synthesis, the samples were rinsed with ultrapure water and then used for the subsequent NO_3_RR experiments.

### Electrolyte preparation for nitrate reduction

The electrolyte used for nitrate reduction experiments is an aqueous solution of 0.1 M Na_2_SO_4_ (anhydrous, 99.99%, Suprapur) +8 mM NaNO_3_ (Sigma-Aldrich, ReagentPlus, ≥99.0%).

### Operando EC-TEM

The EC-TEM experiments were performed in a Thermo Fisher Scientific 300 kV Titan TEM operated in STEM mode with an electron probe current of ∼220 pA. The liquid cell holder used is a Hummingbird Scientific Bulk Liquid Electrochemistry TEM holder with a platinum counter-electrode and a Ag/AgCl (3 M KCl) reference electrode. The EC-TEM top and bottom chips for the cells are produced by Hummingbird Scientific and both have 50-nm-thick silicon nitride membrane windows. Bottom chips with 250 nm spacers are used for these experiments. After cell assembly, the holder was then connected to a Biologic SP-200 potentiostat for electrochemistry experiments. The potentials were measured against a miniature Ag/AgCl reference that is integrated within the holder.

During cell assembly, the TEM holder was prefilled with 0.1 M Na_3_SO_4_ + 8 mM NaNO_3_ solution to fill the entire fluid path with electrolyte. After loading into the TEM, electrolyte was further pumped through the holder at a flow rate of 1.25 ml min^−1^ using a syringe filled with 0.1 M Na_3_SO_4_ + 8 mM NaNO_3_ and a syringe pump. Linear sweep voltammetry from −0.5 to −1.1 V_AgAgCl_ (repeated twice) was first performed at a scan rate of 15 mV s^−1^ to determine the onset potential for the NO_3_RR and to ensure that the applied potential was consistent between experiments. These measurements were then followed up by chronoamperometry measurements for up to 2 h at −0.2, −0.3, −0.4, −0.5 and −0.6 V_RHE_. A new catalyst specimen and fresh electrolyte were used at each applied potential and electrolyte condition.

For these EC-TEM experiments, in situ imaging was always performed under conditions with electrolyte in the cell, as determined from the image contrast. The electron flux was also maintained at 1.75 *e*^−^ Å^−2^ s^−1^ and below at all times to minimize electron-beam-induced artefacts. The acquired images have an image size of 1,024 × 1,024 pixels. During intermittent imaging, the images were acquired every 15 min with the electron beam blanked in between. The image segmentation for the EC-TEM movies was performed using built-in functions and scripting in MATLAB (see Supplementary Note 4 for details).

### NO_3_RR product analysis and detection

The electrochemistry experiments for product analysis and ex situ imaging were conducted using an Autolab potentiostat (PGSTAT 302N) and a custom-made H-type electrochemical cell, in which the cathodic and anodic compartments were separated by an anion-exchange membrane (Selemion AMV, AGC). The counter-electrode is a platinum gauze (MaTecK, 3,600 mesh cm^−2^) and the reference electrode is a leak-free Ag/AgCl electrode (LF-1, Alvatek, potential 0.198 V versus standard hydrogen electrode). The anodic compartment (with counter-electrode) was filled with 18 ml 0.1 M Na_2_SO_4_ electrolyte; the cathodic compartment (with the working electrode) was filled with 18 ml 0.1 M Na_2_SO_4_ electrolyte + 8 mM NaNO_3_. The anodic and cathodic solutions were deaerated before the experiments by continuously bubbling argon (grade 6.0, 99.9999%) with a 20 ml min^−1^ flow rate (Bronkhorst). Linear sweep voltammetry performed at a scan rate of 5 mV s^−1^ was again used to verify the applied potential, and the samples were maintained at constant potential (chronoamperometry) for 2 h for product distribution analysis. A constant argon flow (10 ml min^−1^) was used to maintain the inert atmosphere during chronoamperometry.

An ultraviolet–visible spectrometer (Agilent Cary 60) was used to detect and quantify the amounts of ammonia and nitrite in the electrolyte according to procedures previously established in the literature^48,49^. The liquid electrolyte was first diluted to match the suitable detection range for spectrophotometric analysis of each analyte, and then the sample absorbance was measured in the range 400–800 nm.

The indophenol blue method was used for the determination of NH_3_ (refs. ^48,49^), and a commercial nitrite test kit (photometric 0.002–1.00 mg l^−1^ NO_2_-N, 0.007–3.28 mg l^−1^ NO_2_^−^, Spectroquant, Merck) was used for nitrite quantification. For the latter, 3 ml of the diluted electrolyte was added to a glass vial containing 35 mg of white powder from the kit. Details on quantification can be found in Supplementary Note 2.

### *Ex situ* TEM and SEM measurement

The ex situ TEM imaging was also performed with the Thermo Fisher Scientific 300 kV Titan TEM for before-and-after reaction comparisons. In both cases, the EC-TEM chips were inspected in the TEM using a Hummingbird Scientific Tomography holder. For after-reaction analysis, the EC-TEM chips were first rinsed in ultrapure water after they were disassembled from the EC-TEM holder, and then immediately transferred into the TEM to minimize air exposure. The ex situ SEM imaging of the bulk samples was performed using a Thermo Fisher Scientific Apreo SEM.

### Operando EC-TXM measurement

Operando EC-TXM experiments were conducted at the U41-TXM beamline in BESSY II (Berlin, Germany). The beam size was 26 µm × 26 µm with a nominal resolution of 20 nm. The image stacks were collected using a charge-coupled device detector at 1,340 pixel × 1,300 pixel and an exposure time of 1 s per energy. A 10 nm monochromator slit was used. The intensity of the incident radiation was monitored and adjusted to have a photon count constant (∼15,000 counts per pixel) at the background area (no specimen) when liquid is fully filled. Image stacks were acquired as the beam energies were scanned from 926 to 965 eV, which encompassed both copper L_3_ and L_2_ edges.

The Hummingbird Scientific electrochemistry holder was also used for the operando measurements and the applied potential was controlled with a Biologic potentiostat. The reference electrode was a platinum pseudo-reference on the chip and the counter-electrode was platinum. The reference potential was then calibrated against an external Ag/AgCl electrode to ensure that a potential comparable to the EC-TEM experiments was applied.

Details regarding the data processing, including accurate alignment, background subtraction, data normalization, spectra averaging and LCF of the hyperspectral images can be found in Supplementary Note 2.

### Operando XAS

Operando time-resolved X-ray absorption fine-structure spectroscopy (XAFS) experiments at the copper K edge (8,979 eV) were performed at the P64 beamline of the PETRA III synchrotron (Hamburg,Germany) in quick XAFS mode. The intensity of the incident radiation was monitored by a gas ionization chamber filled with pure nitrogen. Additional ionization chambers were used to acquire spectra of a copper foil in transmission mode for calibration purposes at the beginning of each quick XAFS scan. The beam size was less than 2 mm × 2 mm. The XAS data were collected in fluorescence mode using a passivated implanted planar silicon detector at rates of one spectrum per second and one spectrum per 5 s.

We used a home-made single-compartment electrochemical cell^33^ for these operando XAS experiments. Argon was flowed into the gas compartment at 10 ml min^−1^. The Cu_2_O cubes were prepared on a carbon paper electrode and the 0.1 M Na_2_SO_4_ + 8 mM NaNO_3_ electrolyte was continuously circulated through the cell using a double-channel peristaltic pump. The applied potential was controlled with a Biologic potentiostat.

Data extraction and calibration were performed using the JAQ software of the P64 beamline. Further data processing and analysis of the XANES spectra were performed according to the procedures described previously in ref. ^50^.

### Operando Raman measurement

The operando Raman experiments were performed using a Renishaw (InVia Reflex) confocal Raman microscope and a water immersion objective with a long working distance (Leica Microsystems; 63×; numerical aperture, 0.9) was chosen. The objective was protected from the electrolyte by a Teflon film (DuPont; film thickness, 0.013 mm). Then, a drop of water was used to drive away the air between the film and the objective to match the refractive index to ensure efficient excitation and collection of the Raman signal.

The electrochemical measurements were performed in a home-built spectroelectrochemical cell made of Teflon and controlled by a Biologic SP-240 potentiostat. The cell was equipped with a reference electrode (leak-free Ag/AgCl, Alvatek), a counter-electrode (platinum ring), and a working electrode with the catalyst electrodeposited on glassy carbon. A 15 ml argon-purged 0.1 M Na_2_SO_4_ + 8 mM NaNO_3_ solution was used as an electrolyte. For the experiments on glassy carbon described in Fig. 5, we used a 785 nm laser with 0.1% laser power (0.36 mW). During the experiment, the Raman spectra were acquired every 5 min over 1 h of reaction.

## Online content

Any methods, additional references, Nature Portfolio reporting summaries, source data, extended data, supplementary information, acknowledgements, peer review information; details of author contributions and competing interests; and statements of data and code availability are available at 10.1038/s41563-024-02084-8.

## Supplementary information

Supplementary Information Supplementary Figs. 1–10, Notes 1–6 and references.

Supplementary Video 1 EC-TEM movie describing the structural changes in the Cu_2_O cubes during linear sweep voltammetry from -0.5 to -1.2 V_AgAgCl_ in 0.1 M Na_2_SO_4_ + 8 mM NaNO_3_ (pH 7, NO_3_RR conditions). -0.5 V_AgAgCl_ converts to 0.1 V_RHE_ and -1.2 V_AgAgCl_ converts to -0.6 V_RHE_. The recording rate of the movie was 1 frame per second. 10 frames were averaged to create one frame of the movie. The movie playback rate is ×200 times real time. The electron flux was 1.7 e- Å^-2^ s^-1^.

Supplementary Video 2 EC-TEM movie describing the structural changes in the Cu_2_O cubes during linear sweep voltammetry from -0.5 to -1.2 V_AgAgCl_ in CO_2_^−^saturated 0.1 M KHCO_3_ (pH 6.8, CO_2_RR conditions). -0.5 V_AgAgCl_ converts to 0.1 V_RHE_ and -1.2 V_AgAgCl_ converts to -0.6 V_RHE_. The recording rate of the movie was 1 frame per second. 10 frames were averaged to create one frame of the movie. The movie playback rate is ×200 times real time. The electron flux was 1.7 e- Å^-2^ s^-1^.

Supplementary Video 3 EC-TEM movie describing the structural changes in the Cu_2_O cubes over 2 hours of chronoamperometry at -1.0 V_AgAgCl_ in 0.1 M Na_2_SO_4_ + 8 mM NaNO_3_. -1.0 V_AgAgCl_ converts to -0.4 V_RHE_. The recording rate of the movie was 1 frame per second. 10 frames were averaged to create one frame of the movie. The movie playback rate is ×200 times real time. The electron flux was 1.7 e- Å^-2^ s^-1^.

Supplementary Video 4 EC-TEM movie describing the structural changes in the Cu_2_O cubes over 2 hours of chronoamperometry at -1.1 V_AgAgCl_ in 0.1 M Na_2_SO_4_ + 8 mM NaNO_3_. -1.1 V_AgAgCl_ converts to -0.5 V_RHE_. The recording rate of the movie was 1 frame per second. 10 frames were averaged to create one frame of the movie. The movie playback rate is ×200 times real time. The electron flux was 1.7 e- Å^-2^ s^-1^.

## Data Availability

The authors declare that the data supporting the findings of this study are available within the paper and its Supplementary Information files. The raw data files generated over the course of this study are available from the corresponding authors upon reasonable request.
