# Supplementary material for: Revealing catalyst restructuring and composition during nitrate electroreduction through correlated operando microscopy and spectroscopy
Source: Nat Mater. 2025 Jan 24;24(5):762–9. doi: 10.1038/s41563-024-02084-8 (PMC12048347; doi:10.1038/s41563-024-02084-8)
Supplement: Supplementary file 1 — Supplementary Figs. 1–10, Notes 1–6 and references. [file 41563_2024_2084_MOESM1_ESM.pdf]

nature materials

Supplementary information https://doi.org/10.1038/s41563-024-02084-8

Revealing catalyst restructuring and composition during nitrate electroreduction through correlated operando microscopy and spectroscopy

In the format provided by the authors and unedited

Supplementary Information

Revealing Electrocatalyst Restructuring and Redox Stability During Reaction with Correlated Operando Microscopy and Spectroscopy

Table of Contents

Table of Contents	Page
Supplementary Figures 1-10	1-10
Supplementary Note 1	11-13
Supplementary Note 2	14-15
Supplementary Note 3	16-18
Supplementary Note 4	19-20
Supplementary Note 5	21-24
Supplementary Note 6	25
References	26-27

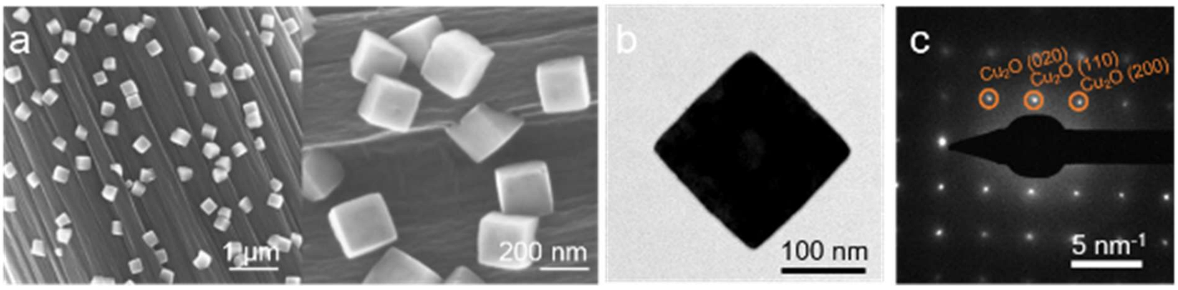

Supplementary Figure 1. Electron microscopy images of as-prepared Cu_2O cubes. (a) *Ex-situ* scanning electron microscopy (SEM), (b) transmission electron microscopy (TEM) and (c) selected area electron diffraction (SAED).

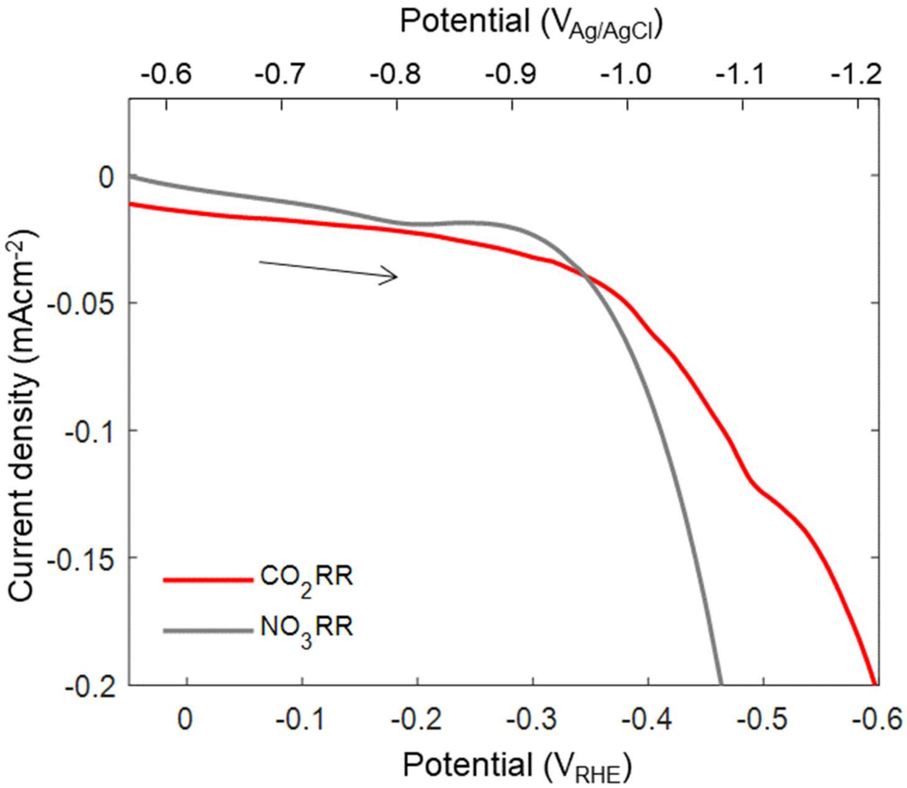

Supplementary Figure 2 Linear sweep voltammetry (LSV) of Cu_2O catalysts under NO_3RR and CO_2RR Conditions: The electrolyte for NO_3RR is 0.1 M Na_2SO_4 + 8 mM $NaNO_3$ (pH 7, grey) and for CO_2RR is CO_2-saturated 0.1 M $KHCO_3$ (pH 6.8 red). All applied potentials are measured against the Ag/AgCl reference electrode and then converted to reversible hydrogen electrode (RHE) using the Nernst equation.

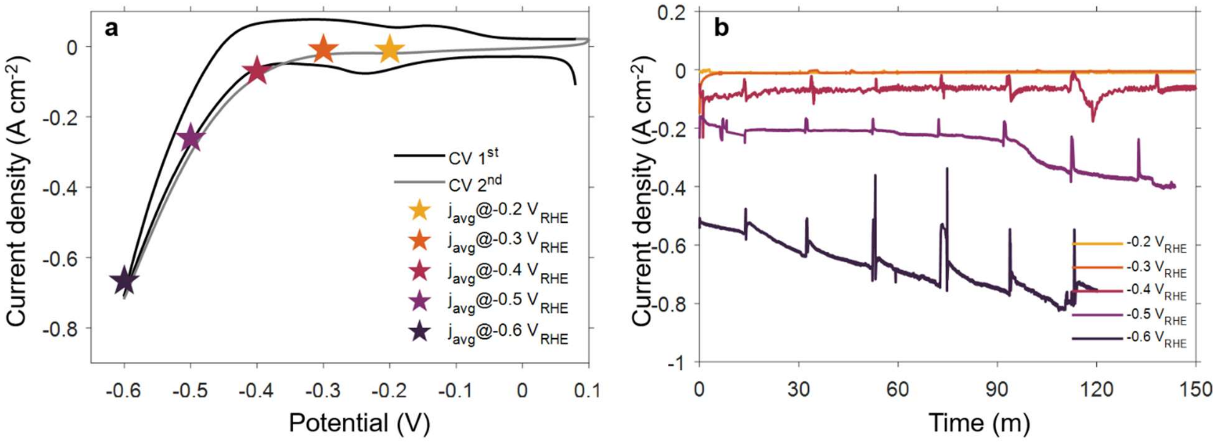

Supplementary Figure 3. Current density profile during the electrolysis in EC-TEM. (a) One and half cyclic voltammetry (CV) marked with the average current density (j_{avg}) at each potential (-0.2, -0.3, -0.4, -0.5, and -0.6 V_{RHE}) in 0.1 M Na_2SO_4 + 8mM $NaNO_3$. (b) Chrono-amperometry at each potential (-0.2, -0.3, -0.4, -0.5, and -0.6 V_{RHE}) over 2 h of measurement in 0.1 M Na_2SO_4 + 8mM $NaNO_3$. The current spikes were due to the exchange of the electrolyte-containing syringes during circulation with a syringe pump. The measurements are referenced to a Ag/AgCl reference electrode and then converted to RHE using the Nernst equation.

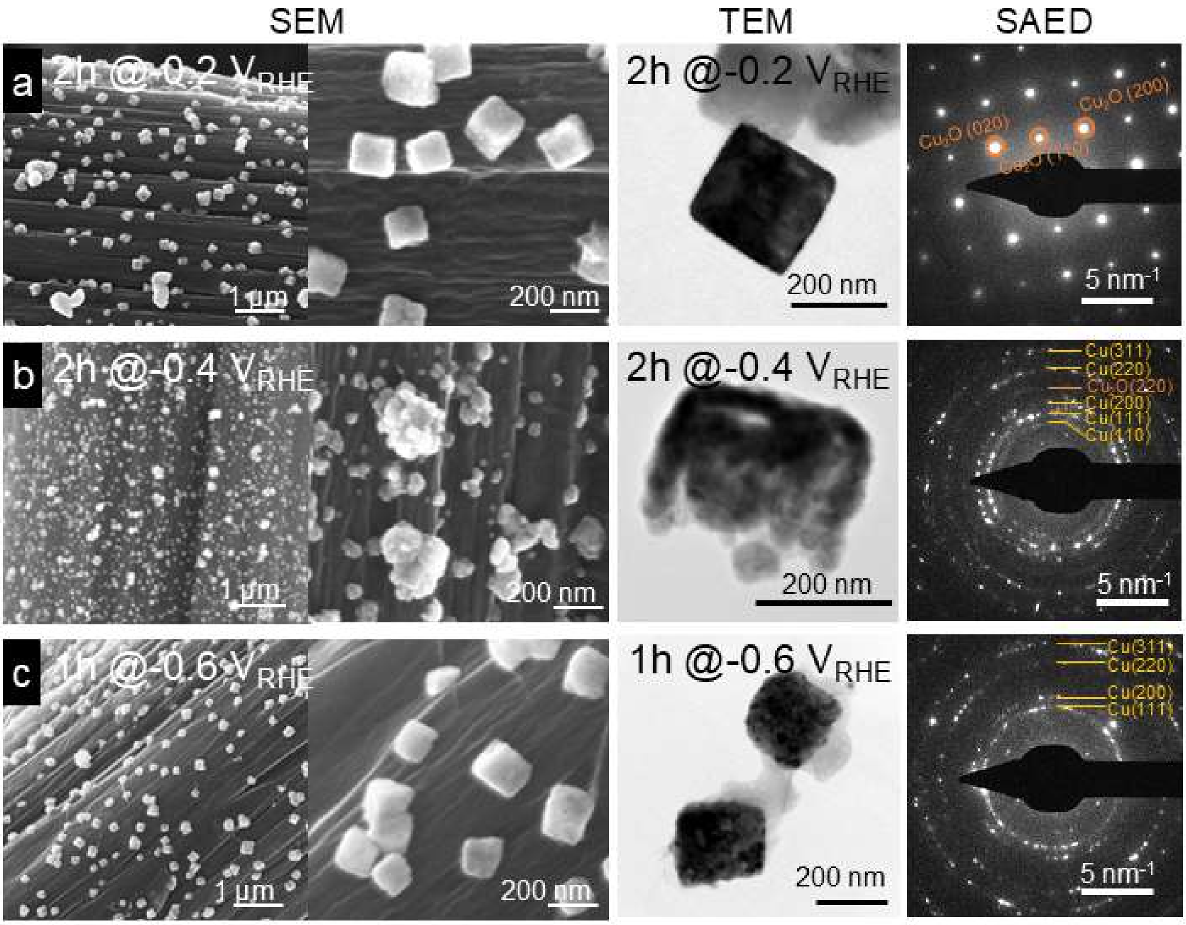

Supplementary Figure 4. Cu_2O cubes after NO_3RR in the H-type cell. Ex-situ SEM, TEM and SAED images of cubes (a) after 2 h of reaction at -0.2 V_{RHE}, (b) after 2 h of reaction at -0.4 V_{RHE} and (c) after 1 h of reaction at -0.6 V_{RHE}. The postmortem analysis was performed immediately after the respective experiments to minimize re-oxidation artifacts from air exposure. The SEM images (left two columns) show the initially cubic pre-catalysts turning into the particulate aggregates at -0.4 V_{RHE}, but remain somewhat cubic after reactions at -0.2 V_{RHE} and -0.6 V_{RHE}. Electron image and diffraction of samples extracted from the carbon paper (right two column) show that the cubes after reaction at -0.2 V_{RHE} were still Cu_2O, whereas the newly formed NPs and the remnant cubes after reactions at -0.4 V_{RHE} and -0.6 V_{RHE} respectively were largely metallic Cu, confirming that the reduction of Cu_2O to Cu only occurred at potentials below -0.6 V_{RHE}.

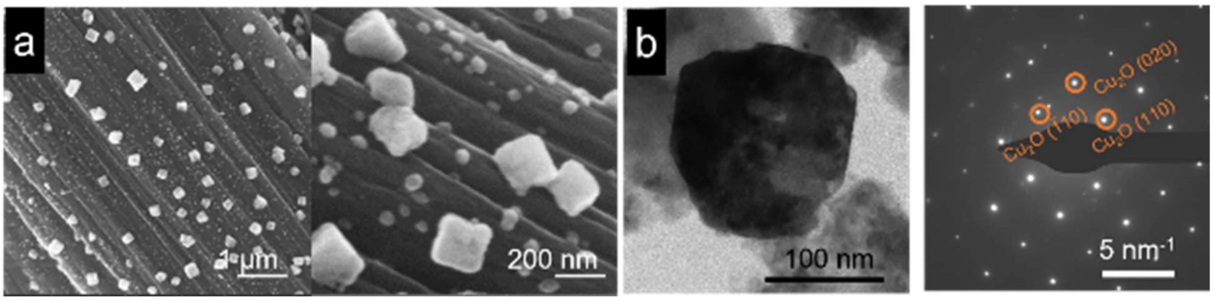

Supplementary Figure 5. Partially dissolved cubes exhibiting the Cu_2O structure after 30 min of reaction at -0.4 V_{RHE} in 0.1 M Na_2SO_4 + 8mM $NaNO_3$. (a) *Ex situ* SEM images showing the remnant cubes and the re-deposited nanoparticles on carbon paper. (b) Post-mortem analysis on the EC-TEM: TEM (left) and SAED (right) show that the partially dissolved cube remains as Cu_2O. The samples were rinsed and dried at the end of the experiment and then inspected immediately.

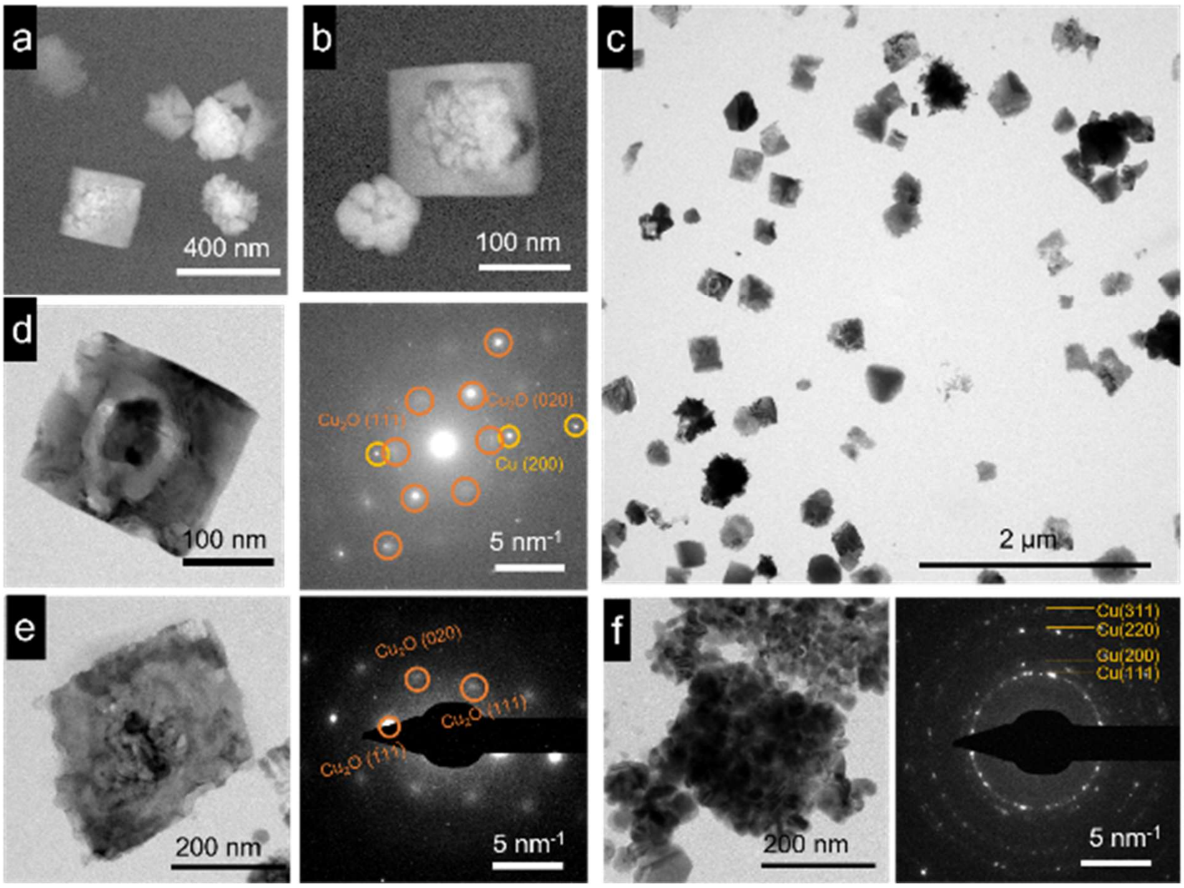

Supplementary Figure 6. Metallic and oxidic phases in Cu catalysts seen in *in situ* and postmortem imaging after 2 h of reaction at -0.4 V_{RHE} in 0.1 M Na_2SO_4 + 8mM $NaNO_3$. *In situ* STEM images of (a-b) random locations on the working electrode at open circuit potential after reaction. (c) Low magnification image shows an area having partially dissolved cubes and re-deposited particles. (d) shows TEM (left) and SAED (right) of a partially dissolved cube, which exhibits both, a metallic Cu and a Cu_2O phase. (e) shows TEM (left) and SAED (right) of a partially dissolved cube, which still has Cu_2O phase. (f) shows TEM (left) and SAED images (right) of the reduced cube and aggregates of re-deposited nanoparticles attached to the cube. Both the cubes and the nanoparticle aggregates are metallic. The samples were rinsed and dried at the end of the experiment and then inspected immediately.

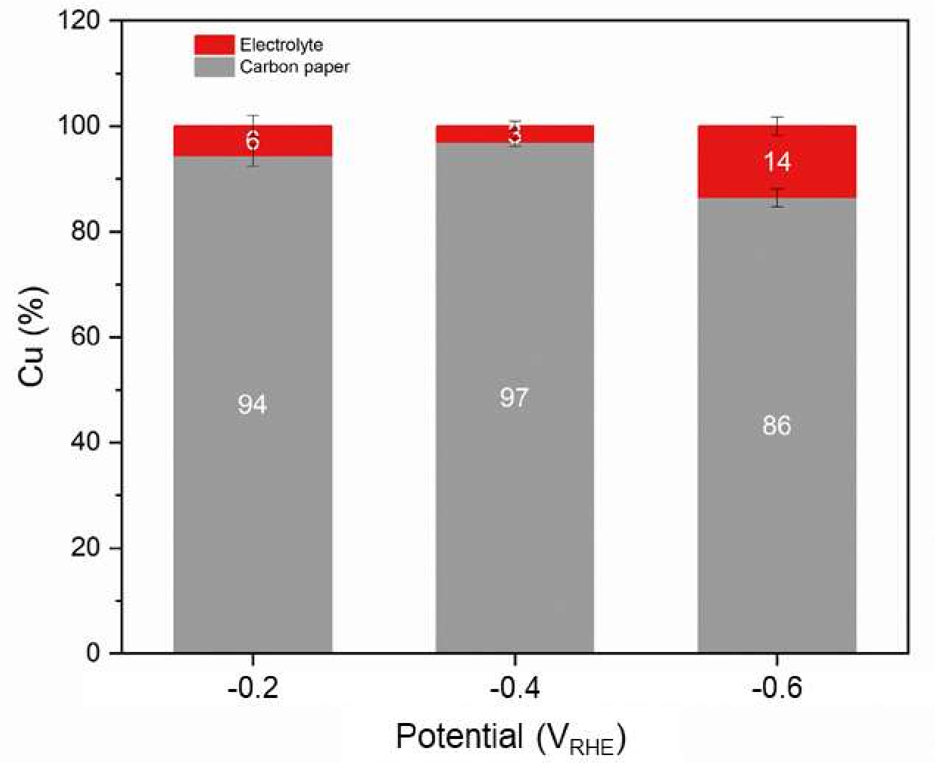

Supplementary Figure 7. Inductively coupled plasma mass spectrometry (ICP-MS) of the reacted electrolyte and sample. The ICP-MS data quantifies the Cu content (%) in the electrolyte (red) and that remains on the carbon paper electrode (gray) after NO_3RR reaction in 0.1 M Na_2SO_4 + 8 mM $NaNO_3$ electrolyte at each potential. The reaction time was 2 h for –0.2 V_{RHE} and –0.4 V_{RHE} and 1 h for –0.6 V_{RHE}. The initial loading of Cu is 15 µg/cm^2. 6 electrolyte samples were extracted from each electrochemical experiment for ICP-MS analysis. The error bars represent the standard deviation of the six samples.

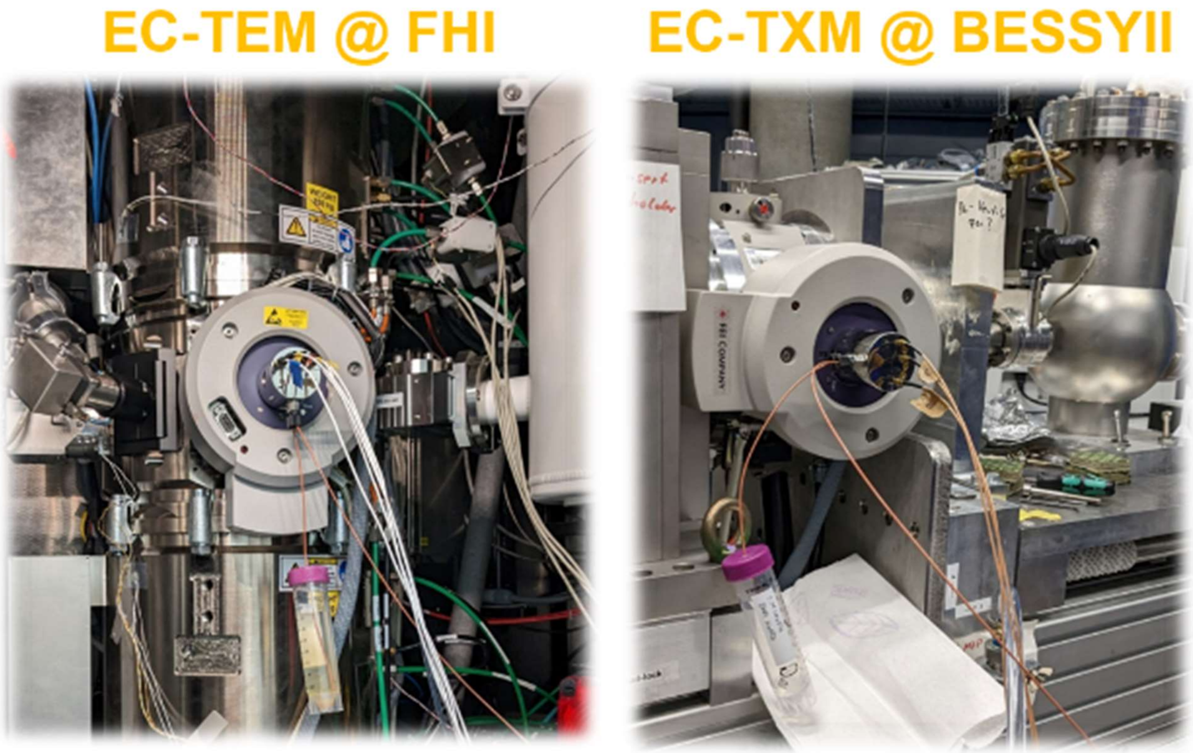

Supplementary Figure 8. The pictures of the holder that is inserted in EC-TEM at Fritz Haber Institute (FHI) and EC-TXM at the U41 beamline at BESSYII.

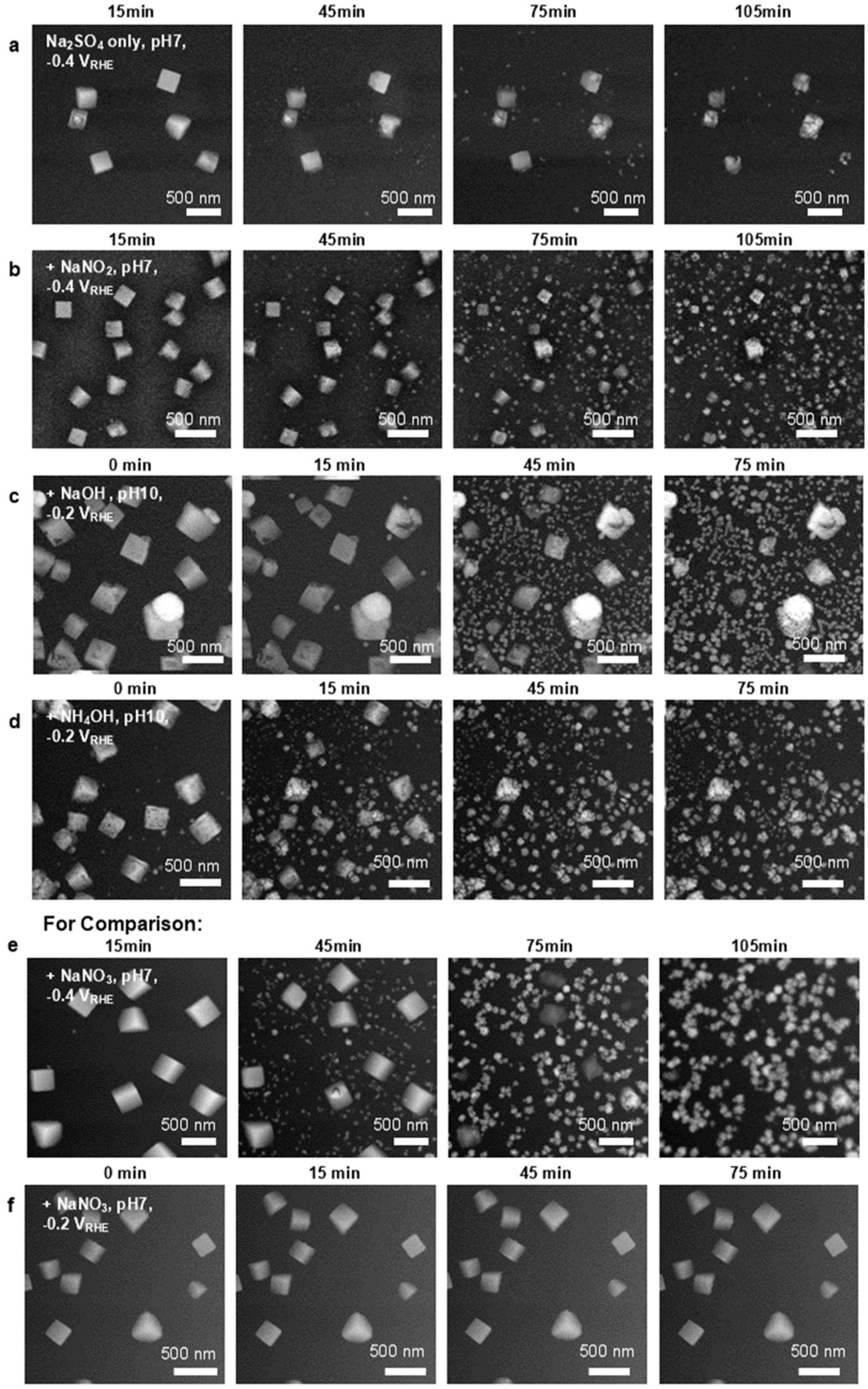

Supplementary Figure 9. Restructuring of Cu_2O cubes in different Na_2SO_4-based electrolytes. EC-TEM image series showing Cu_2O cubes restructuring in 0.1 M Na_2SO_4 with different added chemicals at -1.0 V_{AgAgCl}, i.e. -0.4 V_{RHE} at pH 7 ((a)-(b)) and -0.2 V_{RHE} at pH 10 ((c)-(d)). (a) in 0.1 M Na_2SO_4 only, (b) 0.1M Na_2SO_4 + 8 mM $NaNO_2$, (c) 0.1M Na_2SO_4 where the pH was adjusted to 10 by adding NaOH, (d) Time-series of Cu_2O cubes in 0.1 M Na_2SO_4 + 1 mM NH_3 after 10 min under applied potential in 0.1 M Na_2SO_4 (electrolyte replacement was used here to illustrate the effect of NH_3 addition). For comparison, cubes in 0.1 M Na_2SO_4 + 8mM $NaNO_3$ at -0.4 V_{RHE} and -0.2 V_{RHE} are provided in (e) and (f) respectively.

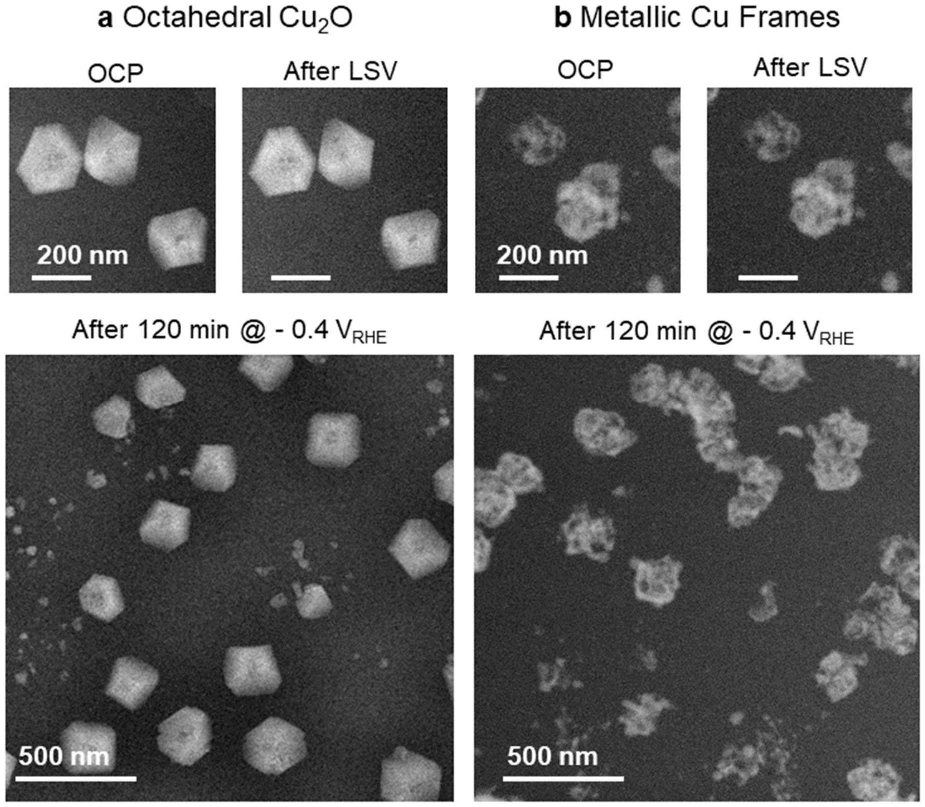

Supplementary Figure 10. EC-TEM images of octahedral Cu_2O and Cu pre-catalysts. Images of (a) octahedrally shaped and (b) metallic Cu frames before and after their initial linear sweep voltammetry measurement and then after 120-min reaction in 0.1 M Na_2SO_4 + 8 mM $NaNO_3$. To form the metallic frames in (b), Cu_2O cubes electrodeposited on the EC-TEM chip were electrochemically pre-reduced in 3-neck round bottom flask. In both cases, we observe minimal restructuring during the LSV. Then, the EC-TEM chip was transferred into the TEM holder and reacted in 0.1 M Na_2SO_4 + 8 mM $NaNO_3$ at -0.4 V_{RHE}.

Supplementary Note 1: Effects of Extended and Continuous Illumination by the Electron Beam

We adopted two imaging protocols to check for the influence that extended electron irradiation during the long durations of the constant potential experiments have on the samples. First, we imaged the area-of-interest continuously but intermittently took images of the catalyst morphology found in another designated area every 15-20 min. Second, we blanked the beam and only looked at the sample after reaction. Even though the continuously imaged catalysts exhibited potential dependent restructuring behavior (Supplementary Videos 3 and 4), we found that we could not avoid accelerated degradation of the Cu_2O cubes under continuous electron beam illumination (Supplementary Figure 11a-b) even when using a low electron flux (1.75 e^{-} A^{-2} s^{-1}). Nonetheless, since the final catalyst morphologies after the sustained reaction in the two separate experiments were similar (Supplementary Figure 11c), the imaging did not appear to alter the evolutionary pathways of the electrocatalysts and their observed morphologies.

With this conclusion, we also looked at the data we acquired with continuously imaging in more detail. Interestingly, contrast changes in the cubes captured during continuous imaging experiments at -0.4 V_{RHE} (Supplementary Videos 3 and Supplementary Figure 12) and -0.5 V_{RHE} (Supplementary Videos 4 and Supplementary Figure 13) suggest that the shrinking Cu_2O cubes may also reduce directly to metal at the late stages of dissolution.

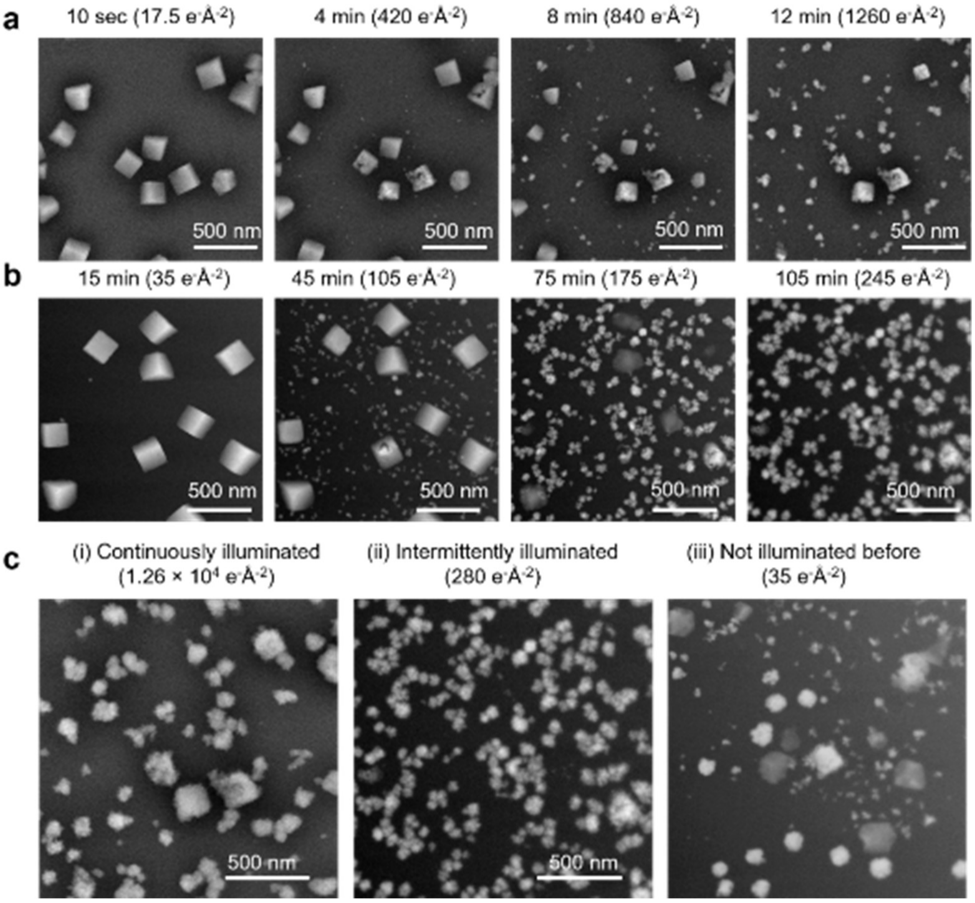

Supplementary Figure 11. Comparison of images acquired using three different imaging modes. The numbers on the top of the images indicate the different reaction times and the accumulated electron dose. (a) Continuously illuminated area at 0, 4, 8, and 12 min of NO_3RR reaction at -0.5 V_{RHE} in 0.1M Na_2SO_4 + 8 mM $NaNO_3$. The electron dose rate was 1.75 $e^-Å^{-2}s^{-1}$ with an acquisition frame rate of 1 fps. The accumulated doses are 17.5, 420, 840, and 1260 $e^-Å^{-2}$ (b) Intermittently illuminated area after 15, 45, 75, and 105 min of NO_3RR at -0.5 V_{RHE} in 0.1M Na_2SO_4 + 8 mM $NaNO_3$, acquired with an electron dose rate of 1.75 $e^-Å^{-2}s^{-1}$. The images were recorded every 15 min. The accumulated doses are 35, 405, 175, and 245 $e^-Å^{-2}$. (c) Three sample areas exposed to (i) continuous illumination, (ii) intermittent illumination, and (iii) an area that was not illuminated. The images were taken with the samples at open circuit potential with the electrolyte retained after 2 h of reaction at -0.5 V_{RHE} in 0.1M Na_2SO_4 + 8 mM $NaNO_3$. The accumulated doses are 1.26×10^4, 280, and 35 $e^-Å^{-2}$, respectively. (i) Under continuous illumination, the cubes dissolve or reduce faster. The sizes of the re-deposited particles are comparable to those in the non-illuminated area. In (ii) the intermittently illuminated area, partially dissolved cubes can still be seen at 75 min, whereas only a few cubes remain in the continuously illuminated area at 12 min in (a). The re-deposited particles in continuously or repeatedly illuminated areas are generally smaller than those of the non-illuminated area and are often interconnected. (iii) In the area not illuminated previously, there were still some cubes-like features.

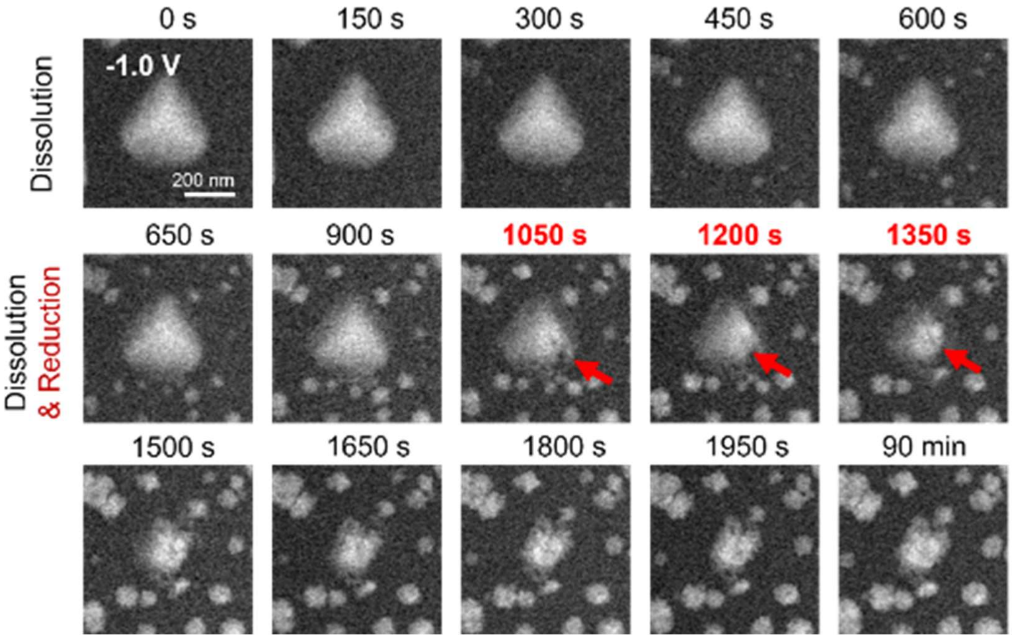

Supplementary Figure 12. Cu_2O cube dissolution and reduction occurring at the same cube captured during NO_3RR at -0.4 V_{RHE} in 0.1 M Na_2SO_4 + 8 mM $NaNO_3$. The frames are extracted from the EC-TEM Supplementary Movie 1 at the indicated reaction times. From 0 to 480 s, cube size is gradually decreasing as dissolution is the dominant process. Between 1050-1350 s, the localized areas with a brighter contrast than the surrounding and the cube fragmentation are observed (marked with the red arrows). We attribute this bright contrast to the metallic phase and so, the contrast change is the sign of direct reduction. After reduction at 1350 s, the residual cube does not dissolve but remain stable with a slight size increase due to re-deposition on its surface.

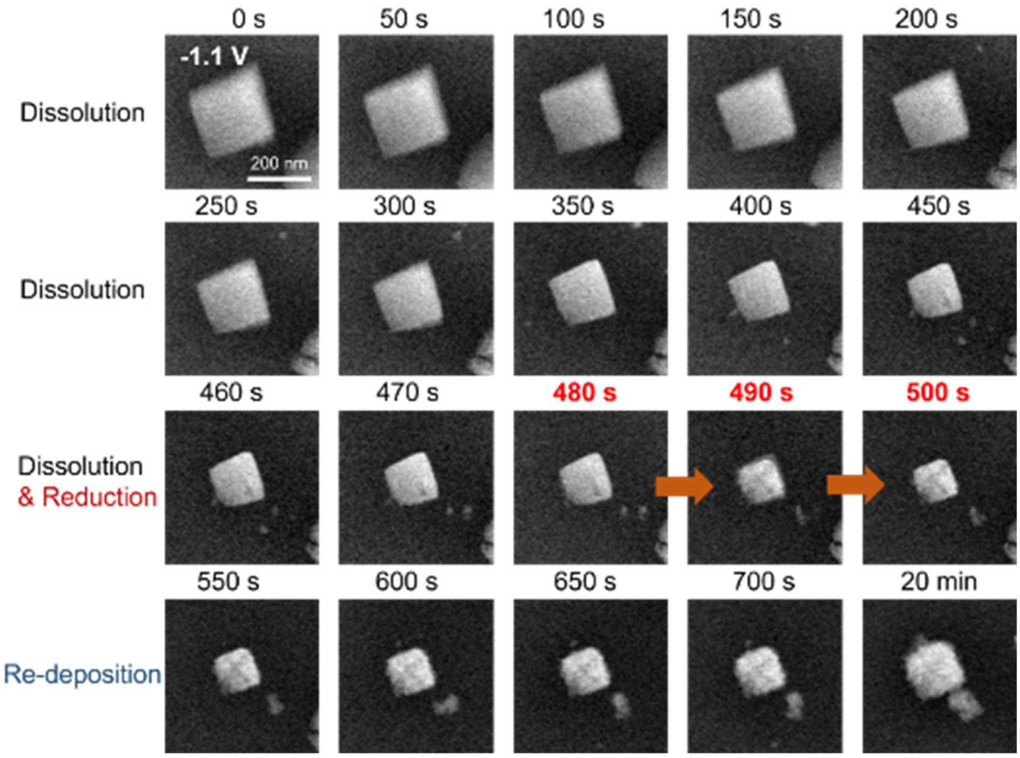

Supplementary Figure 13. Cu_2O cube dissolution and reduction occurring at the same cube captured by EC-TEM during NO_3RR at -0.5 V_{RHE} in 0.1 M Na_2SO_4 + 8 mM $NaNO_3$. The frames are extracted from Supplementary Movie 2 at the indicated reaction times. From 0 to 480 s, the decrease in the cube size indicates that dissolution is the dominant restructuring process. Between 480-500 s, an abrupt size change with inhomogeneous contrast showing inside the shrinking cube can be seen. We attribute this abrupt change to the direct oxide reduction. After reduction at 500 s, the cube size increases over time as Cu particles are re-deposited on the surface of the cube.

Supplementary Note 2: EC-TXM Data Acquisition and Processing

Operando electrochemical transmission X-ray microscopy (EC-TXM) experiment were conducted at U41-TXM beamline[3] in BESSY II (Berlin, Germany) with our Hummingbird Scientific electrochemical holder and identical EC-TEM cells. The beam energy was scanned during experiments over the range of the entire the Cu L-edge (926 eV to 965 eV). The Pt-pseudo reference was calibrated against a bulk Ag/AgCl reference by comparing the redox peak positions and the current densities.

Data analysis: Further data processing, including accurate alignment, background subtraction, data normalization, spectra averaging and linear combination fitting (LCF) of the spectra images were performed. Flatfield correction is applied before getting the background intensity, and then, the image intensity is normalized by the background at each energy. After background normalization, the intensity was again normalized by subtracting the average pre-edge value (E <930 eV) and dividing by the edge jump between the post-edge (935 eV< E < 945eV) and pre-edge. To run LCF, the reference spectra of Cu, Cu_2O and CuO were measured separately as shown in Supplementary Figure 14. We only used the L_3 edge (930 - 945 eV) for fitting to improve the quality of the fit. The raw data at 935 eV and 960 eV of 1st and 2nd scan during the reactions is presented in Supplementary Figure 15.

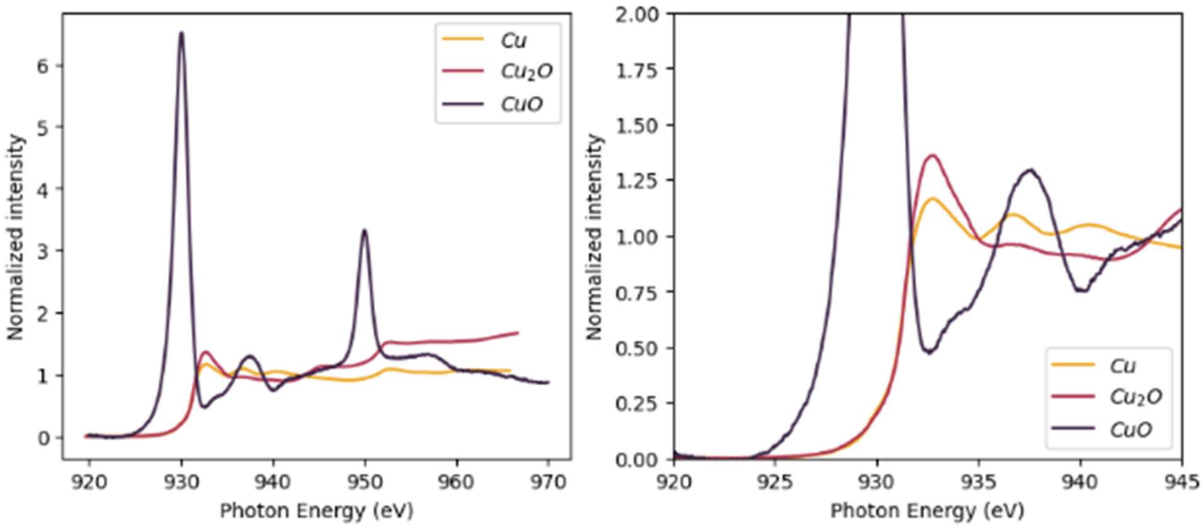

Supplementary Figure 14. Reference Cu L_2 and Cu_{L3} spectra of Cu, Cu_2O, and CuO showing both L_3 and L_2 edges (left) and a magnified plot of the L_3 edge (right)

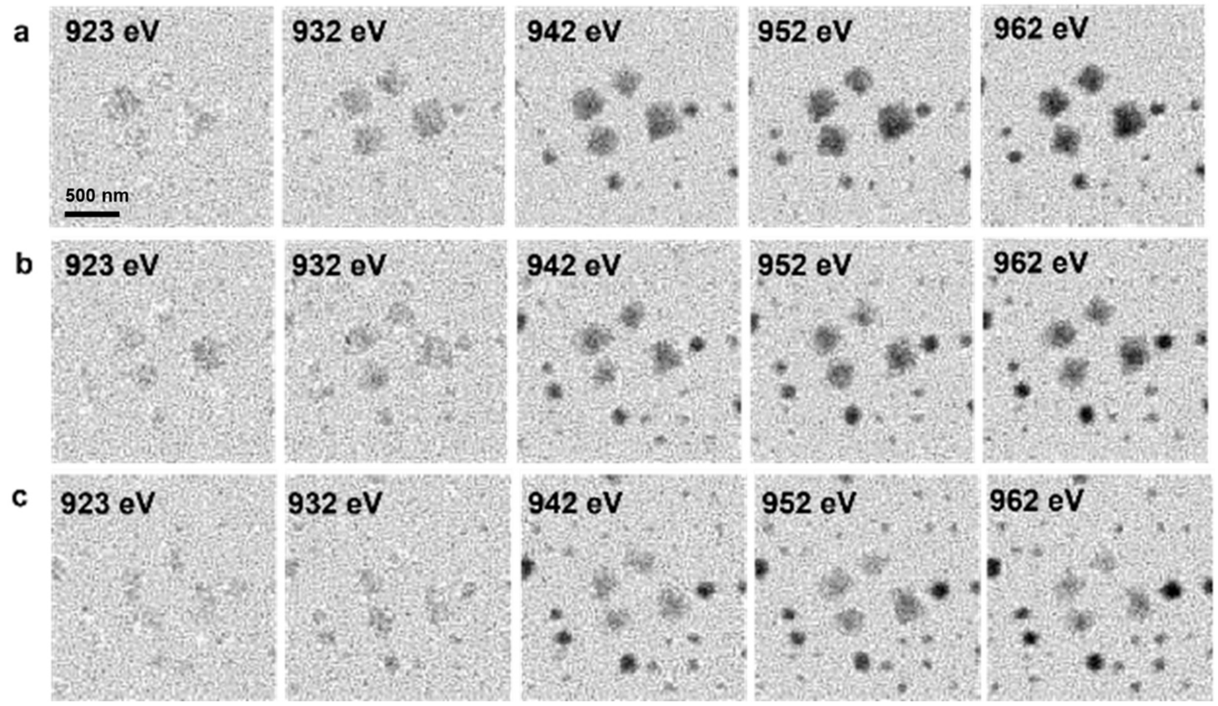

Supplementary Figure 15. Raw data of *operando* EC-TXM. Selected energy slices at 923, 932, 942, 952, at 962 eV of the first scan (a) the second scan (b), and the third scan (c) during NO_3RR at -0.4 V_{RHE} in in 0.1 M Na_2SO_4 + 8mM $NaNO_3$.

Supplementary Note 3: EC-TEM Data Image Processing, Feature Extraction and Analysis of the Re-deposited Particles

The image segmentation for the *in situ* TEM movies was performed by using built-in functions and scripting in MATLAB. The analysis was performed in the following steps: (1) drift correction (2) bandpass filtering, (3) binarization, (4) particle detection, and (5) classification of the cubes and re-deposited particles. The band filter size and binary cutoff threshold were determined by visual inspection. Below is the detailed description of each processing step:

1) Apply a Gaussian and Fourier filter to the image.
2) Convert the grayscale image to a binary image.
3) Identify the cubic particles from the initial frame (we applied size and eccentricity threshold to identify the particles from the random background noise).
4) Label cubes with the center positions and size of boxes (Green box in supplementary Figure 16c).
5) Trace the cubes by tracking and comparing their center position and box size.
6) Identify all the particles: apply size threshold and shape factor (eccentricity and perimeter/area ratio) to filter the random noise (error in).
7) Subtract the labeled cubic area from the area of all particles (Red area in Supplementary Figure 16a).
8) Get the area of the cubic vs non-cubic particles.
9) Convert the area to volume by cube of square root area: (area)^1.5. The assumption here is that all particle shapes are cubic (error in).
10) Summation of the volumes of all particles.
11) Obtain the cube fraction and NP fraction by dividing the volume by the cube volume at the initial frame (t=0 or t=15min for intermittent imaging).
12) Plot the fractions over time (Supplementary Figure 16b).

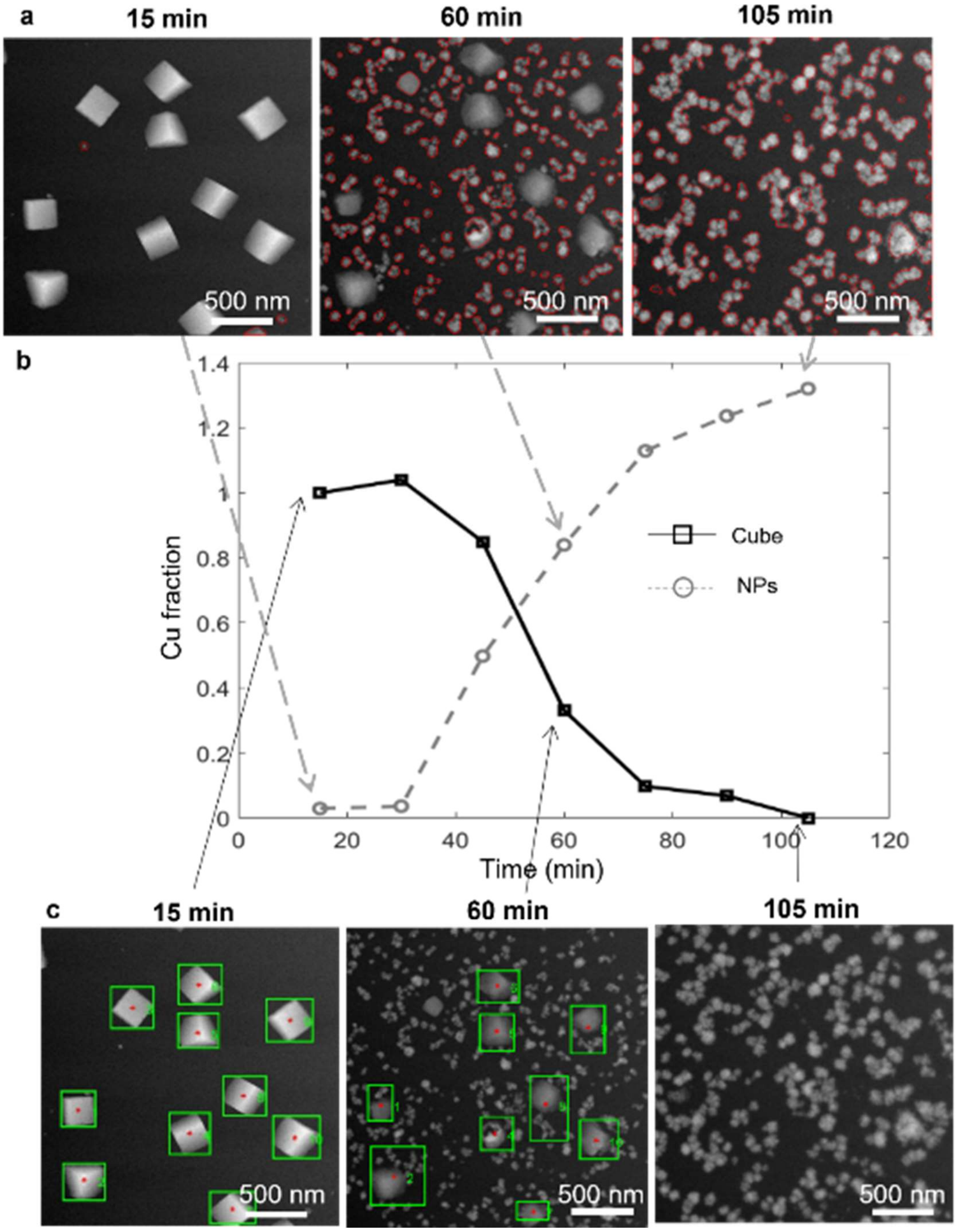

Supplementary Figure 16. (a) An example showing the re-deposited nanoparticles (NPs) being segmented (marked with the red lines) from the image sequence acquired at -0.5 V_{RHE}. (b) Time profile of the cubic and non-cubic NP fraction changes. Each data point represents a cube fraction (square) of the NP fraction (circle) from an image taken at each time. (c) Segmentation of the cubes marked with green boxes.

The cubic fractions extracted from the EC-TEM images as a function of time with this procedure are discussed in the main text and plotted in Figure 4a. The decrease of the cubic fraction leads to a subsequent rise in the fraction of re-deposited NPs (Supplementary Figure 17 below) where the growth rate increases with applied potential, indicating faster deposition of Cu with increased cube dissolution. The slope of the NP fraction, which is equivalent to Cu deposition rate, plateaued at -0.5 V_{RHE} and above. In the case of -0.6 V_{RHE}, we attribute the limited Cu re-deposition at the early stage to a decrease in the concentration of dissolved Cu species caused by the Cu_2O cubes being directly reduced to metallic Cu.

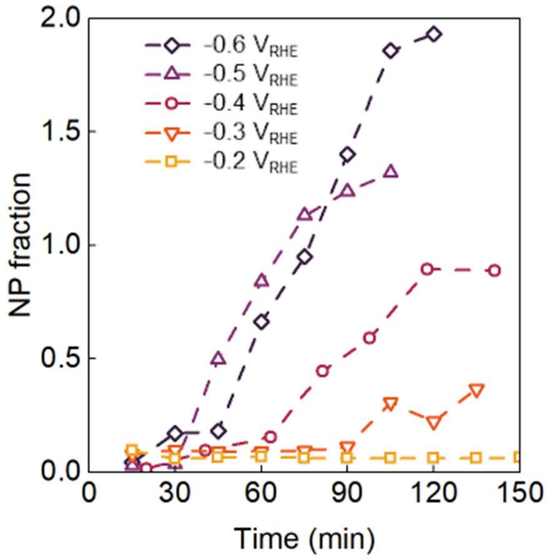

Supplementary Figure 17. Re-deposited nanoparticle (NP) fraction measured from EC-TEM images at the different potentials. In general, the re-deposition rate increases with more negative applied potential up to -0.5 V_{RHE}. At -0.6 V_{RHE}, the onset of visible Cu re-deposition appears to be delayed compared to -0.5 V_{RHE}, which we attribute to a decrease in the concentration of dissolved Cu species caused by the Cu_2O cubes being directly reduced to metallic Cu.

Supplementary Note 4: Complexation Reactions for Cu_2O Dissolution, $Cu(OH)_2$ Precipitation/Dissolution, Cu-ammine Complex Formation and Cu Deposition

Cu-ammine complexes with Cu as a metallic center and a tetragonally bound NH_3 ligand structure[4] are known to be easily formed during the reaction of Cu-hydroxide with NH_3. Candidate complexes include Cu-nitric or Cu-ammine complexes such as $Cu(NO_2)_2(NH_3)_n$, $Cu(NH_3)^{2+}$ $Cu(NH_3)_2^{2+}$, $Cu(NH_3)_3^{2+}$ and $Cu(NH_3)_4^{2+}$.[5–8] The oxidation states and the potentiostatic stability of these complexes also vary with the redox potential applied to the cathode.[9]

The equilibrium reactions governing the dissolution and stabilization of Cu in a NH_3 containing solutions can be obtained from existing literature[5,8]. In principle, there are 12 equilibrium reactions to consider[8], the most relevant ones are described here. Once NH_3 is produced by the nitrate reduction reaction, it also increases the local pH (Equation [1]). NH_3 also has its own acid-equilibrium with water (Equation [2]). $Cu(OH)_2$, which has very low solubility[8], can also form from the reaction of OH^- with Cu_2O and Cu ions (Equation [3] and [4]):

$$NO_3^- + 6H_2O + 8e^- \rightleftarrows NH_3 + 9OH^- \; [1]$$

$$NH_4 + H_2O \rightleftarrows NH_4^+ + OH^- \; [2]$$

$$Cu_2O + 2\,OH^- + H_2O \rightleftarrows 2\,Cu(OH)_2 \; [3]$$

$$Cu^{2+} + 2\,OH^- \rightleftarrows Cu(OH)_2 \; [4]$$

Therefore, solid $Cu(OH)_2$ precipitation is favored when the NH_3 concentration is low. When the NH_3 concentration is high enough, $Cu(OH)_2$ can either forms a soluble complex by reaction with NH_3 as described by Equation [5] [8,10] or by dissolving $Cu(OH)_2$ in the more concentrated alkali, forming $[Cu(OH)_2]^-$ [11] or $[Cu(OH)_4]^{2-}$.

$$Cu(OH)_2 + 4NH_3 \rightleftarrows Cu(NH_3)_4^{2+} + 2OH^- \; [5]$$

These equations show that Cu dissolution and $Cu(OH)_2$ formation/dissolution mainly depend on the NH_3 concentration on the cathodic surface.

As a counter reaction to the dissolution, Cu oxide, Cu hydroxide-ions and Cu-ammine complex ions are electrochemically equilibrated with Cu as shown in Equations [6-10] depending on the redox potential[12];

$$Cu_2O + H_2O + 2e^- \rightleftarrows 2Cu^0 + 2OH^- \; [6]$$

$$Cu(OH)_2^- + e^- \rightleftarrows Cu^0 + 2OH^- \; [7]$$

and

$$Cu(NH_3)_n^{2+} + e^- \rightleftarrows Cu(NH_3)_2^+ + (n-2)NH_3 \; [8]$$

$$Cu(NH_3)_2^+ + e^- \leftrightarrows Cu^0 + 2NH_3 \; [9]$$

$$Cu^0 + Cu(NH_3)_4^{2+} \rightleftarrows 2Cu(NH_3)_2^+ \; [10]$$

In the conventional Pourbaix diagram, the Cu-ammine complex ion is more stable than Cu^{2+} and Cu_2O in pH between 7-12. The complex can stay as Cu(II) in the electrolyte, however, it reduces to metallic Cu at negative potentials[9,13] when they are adsorbed on the electrode surface. The rate of the Cu deposition can be modelled using the Butler-Volmer equation[5]

$$Cu\ deposition\ rate = k^0 \cdot e^{-\alpha \cdot \frac{nF}{RT} \cdot (E-E^0)} \cdot C^s_{Cu(X)_2^+} \quad [11]$$

Where k^0 and α are the intrinsic rate constant and transfer coefficient, and $C^s_{Cu(X)_2^+}$ denotes the Cu-complex ion concentration on the cathodic surface where s is the apparent reaction order and X is either OH^- or NH_3. Here, the equation highlights the key rate determining parameters, the potential difference *(E-E^0)* and $C^s_{Cu(X)_2^+}$, which we could expect faster Cu deposition and depletion of the Cu precursor at more negative potentials. Also, the redox reaction by equation [6] is expected to be faster than the others at more negative potentials because it is a single step process. Therefore, the rate of the restructuring and kinetic pathway is determined by the rate of NH_3 conversion, the ionic stability of the soluble chemical species, and thus, the applied potential.

Supplementary Note 5: *Operando* Raman Spectroscopy Measurements using Cubes Electrodeposited on Carbon Paper

For these experiments, we aim to obtain information about surface adsorbed species and intermediate products in the vicinity of electrode. Therefore, we used a 633 nm laser with stronger laser power (0.1% to 5%) and longer acquisition time (5 s to 10 s), compared to the Raman data in the main text. Carbon paper was used as the electrode substrate to increase the loading of Cu_2O cubes. We first acquired Raman spectra at open circuit potential (OCP), with an average of 3 measurements plotted (Supplementary Figure 18). At OCP, the catalyst shows Raman peaks with obvious features corresponding to Cu_2O (415, 520, and 630 cm^{-1}).

Then, potentials at -0.2 V_{RHE}, -0.4 V_{RHE} and -0.6 V_{RHE} (versus Ag/AgCl) were applied stepwise with a holding duration of 20-30 min at each potential. Supplementary Figure 19-21 shows the full spectra acquired at each potential after different times. We also measured Raman spectroscopy at OCP with an average of 3 measurements after applying -0.6 V_{RHE}. It should be noted that much stronger Raman signals corresponding to Cu_2O features were evidenced after applying -0.2 V_{RHE} for 10 mins (Supplementary Figure 19). The Raman spectra obtained after NO_3RR also showed enhanced signals compared to the OCP spectra of the as-prepared samples (Supplementary Figure 18). This is the result of surface enhanced Raman signals due to surface plasmonic effects of generated nanostructured metallic Cu[14]. In general, a broad band between 400-700 cm^{-1} is seen (expanded in Supplementary Figure 19b, 20b and 21b), which encompasses several Raman bands for Cu_2O, Cu-O and Cu-OH. Although the enhanced signals from Cu_2O are overlapped with other potential band features, a comparison between spectra acquired at different times and potentials still allow us to identify the emergence of a Raman band at ~550 cm^{-1} between the bands at 520 cm^{-1} and 630 cm^{-1} for Cu_2O. Both the band at ~ 475 cm^{-1} we identified on the glassy carbon support and the band at ~550 cm^{-1} had been assigned to OH^- ions adsorbed on Cu under applied cathodic potentials[15], with the former representing bridge-coordinated OH^- and the latter representing top-coordinated OH^- with a hydrogen bonded water molecule. We can therefore infer the presence of surface hydroxide at -0.2 V_{RHE} and -0.4 V_{RHE}. In addition, peak related to Cu-O bending mode (590 cm^{-1}) is observed at -0.2 V and -0.4 V_{RHE}, of which the peak intensity is gradually decreased when applying -0.6 V_{RHE}, consistent with generating more reduced Cu at this condition. These trends are consistent with the catalyst behaviors we had described in the manuscript.

We were also able to identify surface adsorbed species and intermediate products in the vicinity of electrode at different potentials. We show below in Supplementary Table 1, the assignments of these species with their respective Raman bands[16] as highlighted in Supplementary 19a, 20a and 21a. It should be noted here that some bands related to NO_2^-, NO_3^- and NH_3 bands overlaps with the D and G bands of carbon, making their identification more difficult. In -0.2 V_{RHE} and -0.4 V_{RHE}, NH_3, NH_2OH, NO and NO_2^- related peaks were detected. The apparent ratio of the carbon D/G bands under applied potential were also higher than that under OCP condition, suggesting the presence of NO_3^-/NO_2^- related peaks in the carbon D band region. At -0.6 V_{RHE}, the absorbate peaks are weaker compared to those at the -0.2 V and -0.4 V_{RHE}, which may be related to the faster conversion of N intermediates to NH_3. These results are largely consistent with our previous Raman

measurements[16] performed on colloidally synthesized Cu_2O cubes (~30 nm size), while noting that the reactions conditions were not identical (Na_2SO_4 at pH 7 in current study versus Na_2SO_4 at pH 12 in previous study). Nonetheless, this agreement supports the generality of our result.

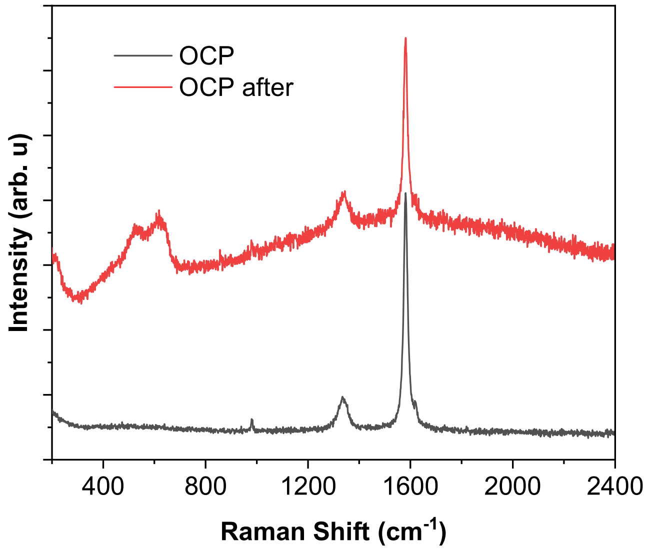

Supplementary Figure 18. Raman Spectra collected at OCP condition before and after NO_3RR. Much stronger Raman signals are due to surface enhancement effect caused by generated metallic Cu during NO_3RR.

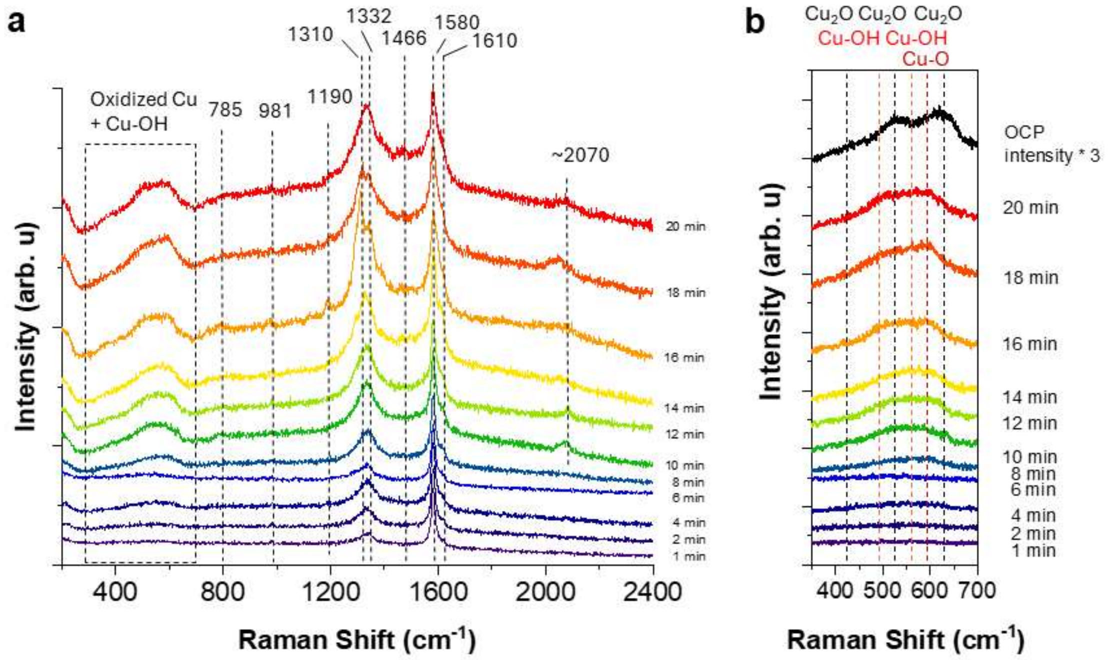

Supplementary Figure 19. Raman Spectra acquired at -0.2 V_{RHE} at different reaction times. (a) Full spectra from 200 cm^{-1} to 2400 cm^{-1} where the Raman bands for the surface species described in Supplementary Table 1 are indicated. (b) expanded plot for the oxidized Cu and Cu-OH/Cu-O region. The spectra of OCP condition after reaction was included for comparison.

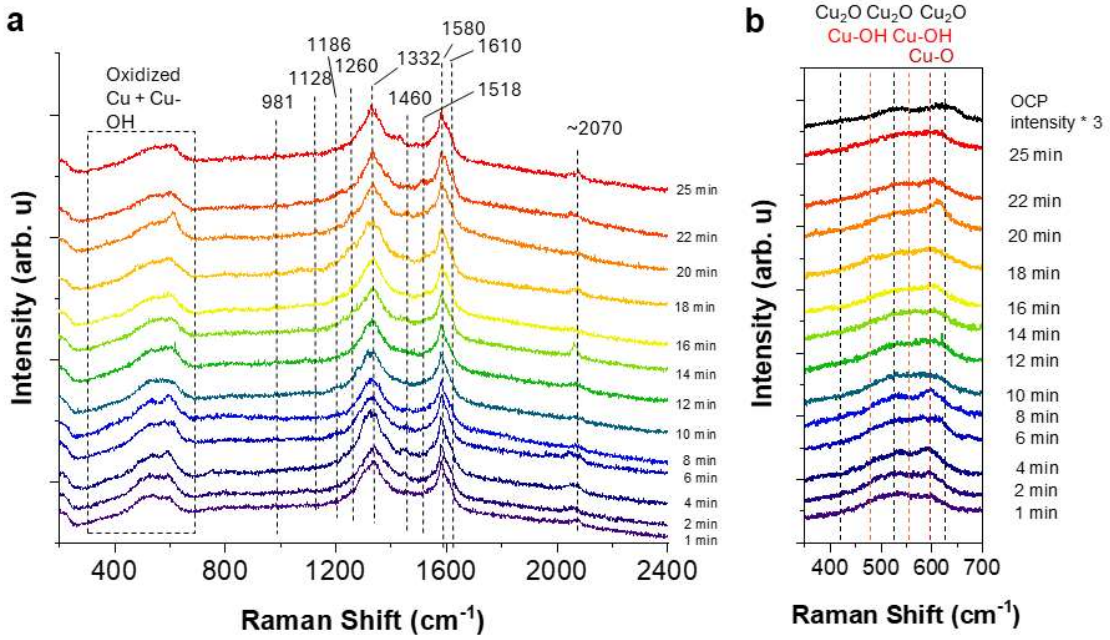

Supplementary Figure 20. Raman Spectra acquired at -0.4 V$_{RHE}$ at different reaction times. The potential was applied to the sample shown in Supplementary Figure 21. (a) Full spectra from 200 cm^{-1} to 2400 cm^{-1} where the Raman bands for the surface species described in Supplementary Table 1 are indicated. (b) expanded plot for the oxidized Cu and Cu-OH/Cu-O region. The spectra of OCP condition after reaction was included for comparison.

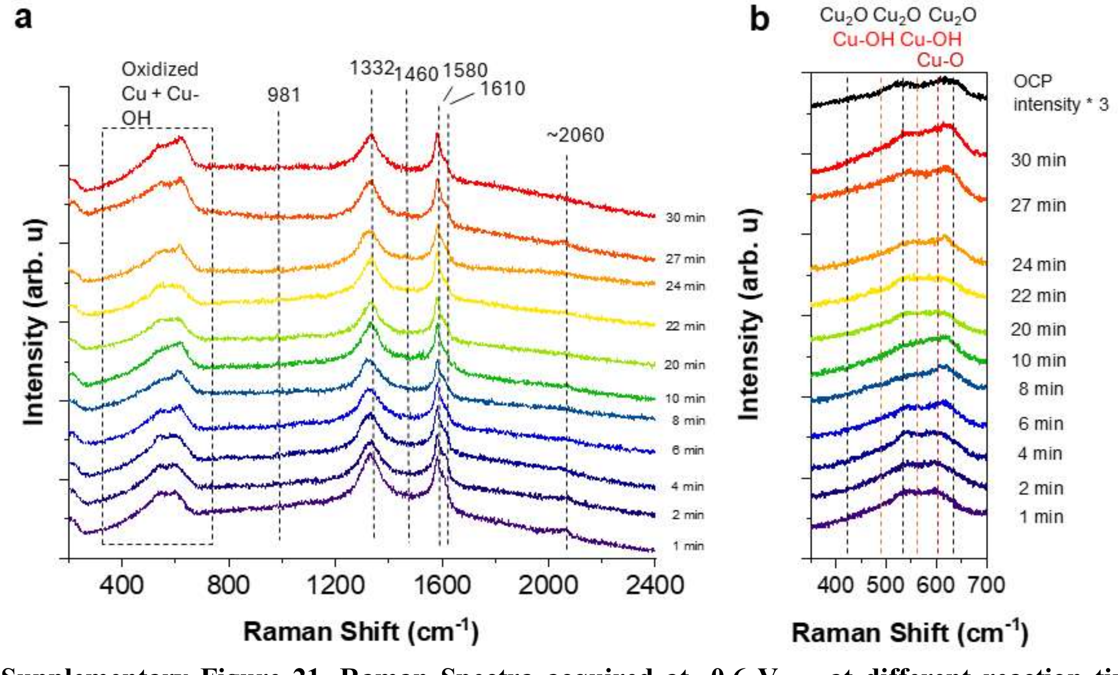

Supplementary Figure 21. Raman Spectra acquired at -0.6 V$_{RHE}$ at different reaction times. The potential was applied to the sample shown in Supplementary Figure 21 and 22. (a) Full spectra from 200 cm^{-1} to 2400 cm^{-1} where the Raman bands for the surface species described in Supplementary Table 1 are

indicated. (b) expanded plot for the oxidized Cu and Cu-OH/Cu-O region. The spectra of OCP condition after reaction was included for comparison.

Supplementary Table 1: Raman bands and their respective assignments

**Raman Band (cm^{-1})**	785	981	1190	1310	1332[a]	1466	1580[b]	1610	~2070
**Assignment**	NO_2^- bending	SO_4^{2-}	antisymmetric N−H bending of NH_2OH	*ONO*⁻ (chelating nitrito)	NO_3^-/ NO_2^- related peaks	ν(T4) antisymmetric NH_2 deformation of NH_4^+	antisymmetric bending vibration of the HNH of NH3	O-H bending of water	*NO

[a] overlaps with D band of carbon
[b] overlaps with G band of carbon

Supplementary Note 6: NO_3RR Product Measurement

Product analysis and detection. UV-Vis spectroscopy (Agilent Cary 60) was used to detect and quantify ammonia and nitrite in the electrolyte after chronoamperometric measurements, following the standard procedures established in previous literature [1,2]. The indophenol-blue method was used for the spectrophotometric determination of ammonia.

Briefly, 500 µL of phenol nitroprusside solution (P6994, Sigma-Aldrich) and 500 µL of alkaline hypochlorite solution (A1727, Sigma-Aldrich) were added to 2 mL of diluted electrolytic sample. The solution was incubated for 30 min at room temperature in the dark before recording the UV-Vis spectrum. Standard solutions with known NH_4Cl (99.998%, Sigma-Aldrich) concentration were used to construct a calibration curve. The faradaic efficiency (FE_{NH3}) and the yield rate (r_{NH3}, mmol h^{-1} cm^{-2}) for ammonia formation were calculated according to (Equation [12]) and (Equation [13]), respectively:

$$FE_{NH3} = \frac{(8F \times c_{NH3} \times V)}{(M_{NH3} \times Q)} \times 100\% \qquad [12]$$

$$r_{NH3} = \frac{(c_{NH3} \times V)}{(M_{NH3} \times A \times t)} \qquad [13]$$

where c_{NH3} is the mass concentration of NH_3(aq) , V is the volume of electrolyte in the cathode compartment, F is the Faradaic constant (96485 C mol^{-1}), M_{NH3} is the molar mass of NH_3, Q is the charge passed during electrolysis, A is the geometric area of the electrode and t is the electrolysis time.

For nitrite (NO_2^-) quantification, 3 mL of the diluted sample were added to a glass vial containing 35 mg of white powder from a commercial nitrite test kit (photometric 0.002–1.00 mg L^{-1} NO_2-N, 0.007–3.28 mg L^{-1} NO_2^-, Spectroquant, Merck). The mixture was then incubated for 10 min at room temperature in the dark before UV-Vis analysis. Analogously to ammonia, a calibration curve was constructed by using standard $NaNO_2$ (99%, for analysis, Sigma-Aldrich) concentrations. For electrocatalytic NO_3RR experiments, the faradaic efficiency (FE_{NO2-}) and the yield rate (r_{NO2-}, mmol h^{-1} cm^{-2}) for nitrite formation was calculated according to Equation [14] and Equation [15], respectively:

$$FE_{NO2-} = \frac{(2F \times c_{NO2-} \times V)}{(M_{NO2-} \times Q)} \times 100\% \qquad [14]$$

$$r_{NO2-} = \frac{(c_{NO2-} \times V)}{(M_{NO2-} \times A \times t)} \qquad [15]$$

where c_{NO2-} is the mass concentration of NO_2^-(aq), V is the volume of electrolyte in the cathode compartment, F is the Faradaic constant (96485 C mol^{-1}), M_{NO2^-} is the molar mass of NO_2^-, Q is the charge passed during electrolysis.

References

1. Andersen, S. Z. *et al.* A rigorous electrochemical ammonia synthesis protocol with quantitative isotope measurements. *Nature* **570**, 504–508 (2019).

2. Wang, Y., Wang, C., Li, M., Yu, Y. & Zhang, B. Nitrate electroreduction: mechanism insight, in situ characterization, performance evaluation, and challenges. *Chem. Soc. Rev.* **50**, 6720–6733 (2021).

3. Rehbein, S., Heim, S., Guttmann, P., Werner, S. & Schneider, G. Ultrahigh-Resolution Soft-X-Ray Microscopy with Zone Plates in High Orders of Diffraction. *Phys. Rev. Lett.* **103**, 110801 (2009).

4. Mazzi, F. *The Crystal Structure of Cupric Tetrammine Sulfate Monohydrate, Cu(NH3),SO,. H20. Acta Cryst* vol. 8 (1955).

5. Giannopoulou, I., Panias, D. & Paspaliaris, I. Electrochemical modeling and study of copper deposition from concentrated ammoniacal sulfate solutions. *Hydrometallurgy* **99**, 58–66 (2009).

6. Schroder, D., Schwarz, H., Wu, J. & Wesdemiotis, C. Long-lived dications of Cu(H2O)2+ and Cu(NH3)2+ do exist! *Chem. Phys. Lett.* **3343**, 258–264 (2001).

7. Ducéré, J. M., Goursot, A. & Berthomieu, D. Comparative density functional theory study of the binding of ligands to Cu+ and Cu2+: Influence of the coordination and oxidation state. *J. Phys. Chem. A* **109**, 400–408 (2005).

8. Johnson, A. R., McQueen, T. M. & Rodolfa, K. T. Species distribution diagrams in the copper-ammonia system: An updated and expanded demonstration illustrating complex equilibria. *J. Chem. Educ.* **82**, 408–414 (2005).

9. Hoar, T. P. & Rothwell, G. P. The potential/pH diagram for a copper-water-ammonia system: its significance in the stress-corrosion cracking of brass in ammoniacal solutions. *Electrochim. Acta* **15**, 1037–1045 (1970).

10. Luo, Q., Mackay, R. A. & Babu, S. V. Copper Dissolution in Aqueous Ammonia-Containing Media during Chemical Mechanical Polishing. *Chem. Mater.* **9**, 2101–2106 (1997).

11. Celante, V. G. & Freitas, M. B. J. G. Electrodeposition of copper from spent Li-ion batteries by electrochemical quartz crystal microbalance and impedance spectroscopy techniques. *J. Appl. Electrochem.* **40**, 233–239 (2010).

12. Ciavatta, L., Ferri, D. & Palombari, R. On the equilibrium Cu2+ + Cu(s) ⇄2Cu+. *J. Inorg. Nucl. Chem.* **42**, 593–598 (1980).

13. Speck, F. D. & Cherevko, S. Electrochemical copper dissolution: A benchmark for stable CO2 reduction on copper electrocatalysts. *Electrochem. commun.* **115**, (2020).

14. Zhan, C. *et al.* Revealing the CO Coverage-Driven C-C Coupling Mechanism for Electrochemical CO2Reduction on Cu2O Nanocubesvia OperandoRaman Spectroscopy. *ACS Catal.* **11**, 7694–7701 (2021).

15. G. Niaura. Surface-enhanced Raman spectroscopic observation of two kinds of adsorbed OH− ions at copper electrode. *Electrochim. Acta* **45**, 3507–3519 (2000).

16. Bai, L. *et al.* Electrocatalytic Nitrate and Nitrite Reduction toward Ammonia Using Cu2O Nanocubes: Active Species and Reaction Mechanisms. *J. Am. Chem. Soc.* **146**, 9665–9678 (2024).
